# Switching to nanonutrients for sustaining agroecosystems and environment: the challenges and benefits in moving up from ionic to particle feeding

**DOI:** 10.1186/s12951-021-01177-9

**Published:** 2022-01-04

**Authors:** Ajay Kumar Bhardwaj, Geeta Arya, Raj Kumar, Lamy Hamed, Hadi Pirasteh-Anosheh, Poonam Jasrotia, Prem Lal Kashyap, Gyanendra Pratap Singh

**Affiliations:** 1 ICAR-Central Soil Salinity Research Institute, Karnal, Haryana 132001 India; 2 Soil and Water Department, Faculty of Agriculture, Cairo University, Giza, 12613 Egypt; 3 National Salinity Research Center, Agricultural Research, Education and Extension Organization, Yazd, 8917357676 Iran; 4 ICAR-Indian Institute of Wheat and Barley Research, Karnal, Haryana 132001 India

**Keywords:** Plant nutrients, Nanotechnology, Fertilizers, Use efficiency, Controlled release, Agriculture

## Abstract

The worldwide agricultural enterprise is facing immense pressure to intensify to feed the world’s increasing population while the resources are dwindling. Fertilizers which are deemed as indispensable inputs for food, fodder, and fuel production now also represent the dark side of the intensive food production system. With most crop production systems focused on increasing the quantity of produce, indiscriminate use of fertilizers has created havoc for the environment and damaged the fiber of the biogeosphere. Deteriorated nutritional quality of food and contribution to impaired ecosystem services are the major limiting factors in the further growth of the fertilizer sector. Nanotechnology in agriculture has come up as a better and seemingly sustainable solution to meet production targets as well as maintaining the environmental quality by use of less quantity of raw materials and active ingredients, increased nutrient use-efficiency by plants, and decreased environmental losses of nutrients. However, the use of nanofertilizers has so far been limited largely to controlled environments of laboratories, greenhouses, and institutional research experiments; production and availability on large scale are still lagging yet catching up fast. Despite perceivable advantages, the use of nanofertilizers is many times debated for adoption at a large scale. The scenario is gradually changing, worldwide, towards the use of nanofertilizers, especially macronutrients like nitrogen (e.g. market release of nano-urea to replace conventional urea in South Asia), to arrest environmental degradation and uphold vital ecosystem services which are in critical condition. This review offers a discussion on the purpose with which the nanofertilizers took shape, the benefits which can be achieved, and the challenges which nanofertilizers face for further development and real-world use, substantiated with the significant pieces of scientific evidence available so far.

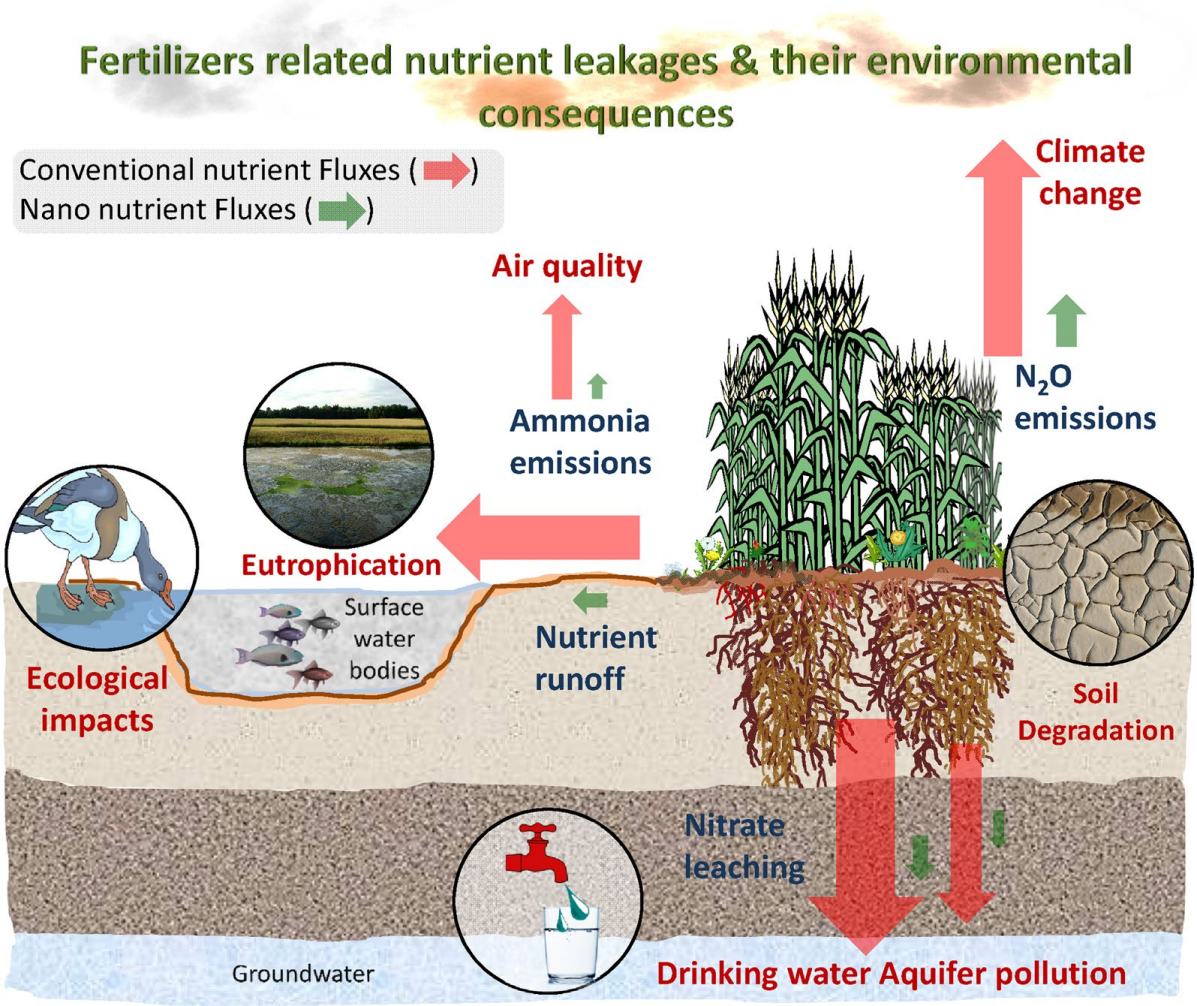

## Background

Agriculture is one of the major sectors that contribute to the self-sufficiency, and economic growth of many countries, worldwide. Several factors hamper the growth of this sector, including fragmentation of land use due to increase in population, a mass exodus of the workforce from farming to other industrial enterprises, and limited availability of natural resources [47]. The use of synthetic fertilizers was very limited till 1913 when the Haber–Bosch process was discovered which resulted in the immense growth in the production and use of nitrogen fertilizers. The green revolution in the 1960s led to the use of intensive farming practices which resulted in a substantial increase in crop yields, and agrochemicals, especially fertilizers, played a significant role [29, 112]. The increase in cropping intensity and fertilizer use undoubtedly played role in alleviating worldwide hunger especially in Asia and Africa but this was not without its side-effects like decreased nutrient use efficiency, micronutrient deficiencies due to overuse of few major nutrients (Nitrogen, phosphorus, potassium), deterioration of soil quality, and very detrimental effects on the surrounding environment. Further, according to a recent report of Food and Agricultural Organization (FAO), the primary natural resources like arable land and water are continuously getting exhausted due to intensive urbanization and societal changes. All these issues have become limitations for the growth of agriculture, in the post-green revolution era [33, 135].

Despite the low uptake and high losses (many times as high as 70% for nitrogen and potassium, and 90% for phosphorus), synthetic chemical fertilization has proved to fulfill the nutrient demands of crops worldwide, to meet the demand for food by the exploding world population which is going to surpass 10 billion by the end of 2050 [40, 154]. The pressure of fulfilling this demand as well as the heavily subsidized cost of synthetic chemical fertilizers, especially macronutrients, are the key reasons for the abusive use of fertilizers by the farmers. The indiscriminate use of fertilizers intensifies environmental damage by abetting the emission of harmful greenhouse gases (e.g. N_2_O), eutrophication of surface water bodies, contamination of groundwater, and distortion of natural biogeochemical cycles. The biased fertilizer application favoring macronutrients has also led to the deficiency of essential micro-nutrients because the food brings to the dining table what it is fed in the field [28, 172]. The imbalances in the soil negatively impact the growth, yield, and nutritional quality of food [144]. Some estimates indicate that around two-thirds of the world’s population, including both developed and developing countries, is facing the risk of deficiency of essential mineral nutrients [9, 154]. At present there are an estimated 720–811 million undernourished people, worldwide, and this figure will reach 840 million by 2030 [45]. The current worldwide scenario in fertilizer use is not only skewed but has also surpassed the boundaries of essentiality and eschewed the sustainability criteria.

Overcoming these daunting challenges of eradicating worldwide hunger as well as saving the planet’s natural fiber calls for an urgent improvement in the technology of nutrient use by improving the nature of fertilizers or by improving their application method, to improve use-efficiency as well as decrease total nutrient use [22, 89, 129]. All improvements need to have a balance of eco-friendliness as well as cost-effectiveness for acceptance by the farming community. In this direction, nanotechnology has opened a good scope for both improvements (increased uptake efficiency and precision application) and therefore has the potential to revolutionize the agricultural sector for agrochemical use [33]. Nanotechnology deals with the designing of ultra-small entities, having at least one dimension in the range of 1–100 nm. These nano-sized entities or materials represent the transition zone between the individual atom or molecules and their bulk materials, and exhibit extraordinary properties as compared to the bulk counterparts in terms of reactivity and other physico-chemical properties [21]. The properties of nanoparticles (NPs) are attributed to their high surface area to volume ratio that enhances their chemical reactivity as well as physical responses. That means less amount of nanomaterial can do the same or even better job than their bulk forms. Like many other sectors, fertilizer research and development has also adopted nanotechnology to overcome the challenges and criticisms faced by the industry as well as by the farming community in terms of indiscriminate natural resource exploitation. In the agricultural sector, nanofertilizers are the most desired output of nanotechnology and has the motivation for controlled and prolonged release of nutrient, in a signal responsive manner (to temperature or pH), or for simply switching to an efficient mode of application (e.g. foliar application) to increase nutrient uptake efficiency and reduce losses. A small amount of nanofertilizer should be sufficient to achieve the required production goal as compared to the conventional fertilizers which need heavy application [172]. Nanofertilizers can enhance nutrient availability to plants, minimize losses of nutrients via leaching, and would have minimal impact on the environment. In totality, the goal is to minimize energy use, reduce environmental losses of nutrients, and do this without losing (or rather improving) crop productivity.

Despite all these perceived advantages, nanofertilizers are also surrounded by contradictions attributed to the conflicting results of some similar formulations on the same or different plant species, and sparse reports of nanotoxicity and genotoxicity of some formulations [78, 81]. Further, based on the unique properties of some of the engineered formulations, the term “nano” is sometimes even associated with a range beyond 100 nm, and up to 1000 nm particle size which is also considered a debatable issue [67]. Added to this, there are awareness groups (ETC and Friends of Earth) that believe in a complete ban on nanoscale formulations for agriculture use until there is full clarity in the regulatory regime in terms of their safety for humans and other dwellers of the natural ecosystems [53, 108]. The conventional (most common) practice of nutrient use is the granular fertilizer application to the soil, wherein nutrient elements upon dissolution of fertilizers are released in ionic forms for plant roots to absorb along with water. Though there are established advantages in the foliar application of NPs, what advantage does a nano-sized nutrient particle provide as the nutrients in the conventional fertilizers are available to the plants in ionic forms after dissolution, and ions have a smaller size than NPs? Changes in the application method to increase efficiency can also have altogether different implications especially if the active ingredients exist in nano-forms (e.g. more energy use for application). The purpose of this review, therefore, is to present in detail most of the aspects of nanofertilizers including the size characteristics (compared to conventional nutrient forms), exploited purposes, novel properties and synthesis techniques, benefits, and possible hindrances in further development. The review also provides an overview of conventional fertilizers viz-a-viz nanofertilizers for nutrient forms, sizes, and behavior in soil environment regarding plant uptake.

## An overview of nutrient requirements of plants

**Fig. 1:**
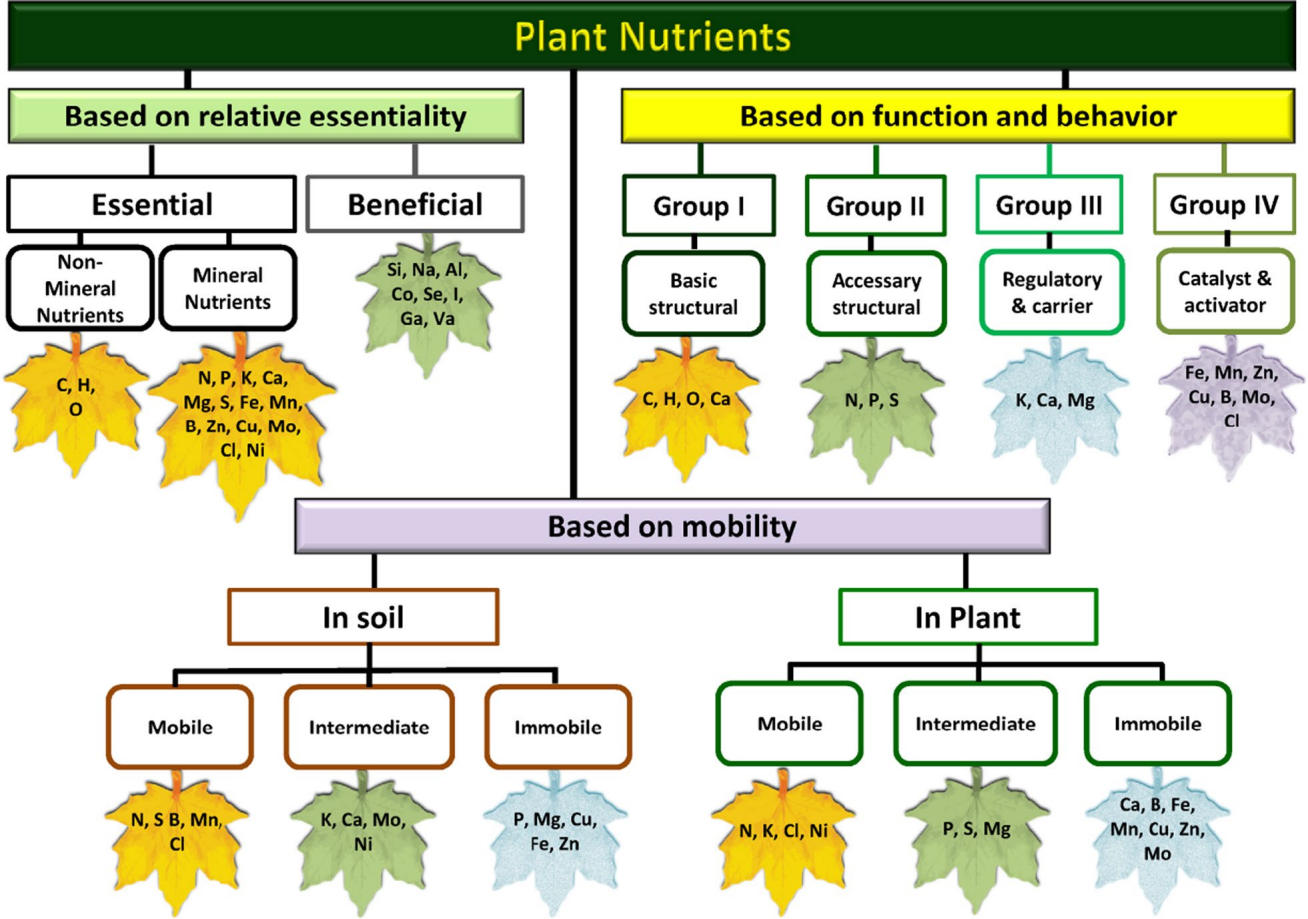
Categories of essential plant nutrients based on the relative essentiality, function, and mobility in soil and plants

Plants require some important elemental nutrients to complete their growth and development over an entire life cycle, and in absence of these nutrients the growth processes are hindered, and development is stunted, both in crop plants and the animals and humans feeding on the produce from these crops (Table 1). These elements in ionic forms are the principal immediate source of nutrients for plants. Justus von Liebig extensively laid the foundation of the importance of mineral nutrients for plant growth, and also established a scientific discipline that led to the introduction and advancement of the concept of mineral fertilizers. An ample amount of work further conducted under this discipline by several other workers established that some mineral elements are absolutely important for plants to complete their lifecycle and metabolic activities. These elements are referred to as “essential elements”, and the term was coined by Arnon and stout in 1939. A criterion was set for essentiality, and based on it 17 elements are now considered essential for plant growth. These essential elements are categorized into two groups including (1) non-mineral elements (consisting of 3 elements including C, H, and O which constitute about 95% of total plant dry weight and are taken up from the atmospheric CO_2_ and soil water, and (2) mineral elements which are taken up mostly from soil (consisting of 14 elements N, P, K, Ca, Mg, S, Fe, Mn, B, Zn, Cu, Mo, Cl, and Ni). The essential mineral elements are taken up in ionic forms from the soil solution. These mineral elements are further categorized into three broad groups including, (i) grouping based on their utilization and absorption by the plants (macronutrient and micronutrient), (ii) based on their physiological function, and (iii) based on their mobility in the phloem. These groups are further categorized into subgroups based on various essentiality criteria (Fig. 1).

**Table 1:** Fertilizer nutrients related deficiency symptoms in plants, and nutritional disorders in animals and humans

Nutrient element	Deficiency symptoms in plants	Nutritional disorders in animals and humans	Ref.
Nitrogen, N	Severe chlorosis, necrosis of leaves, stunted growth, reduced fertilization, and curtailed fruit yield	Protein malnutrition or intestinal malabsorption	[66, 100]
Phosphorus, P	Bluish-green leaves, restricted growth	Vitamin D deficiency, rickets in infants, and osteomalacia in adults	[38, 100]
Potassium, K	Burning along with spotting in leaf margins, reduced crop yield quality of fruit and vegetables	Hypokalemia, risk of cardiovascular diseases, and impaired bone health	[75, 100, 159]
Calcium, Ca	Chlorosis in young leaves	Rickets, osteoporosis. Osteopenia with disturbed metabolic deficiency	
Magnesium, Mg	Chlorosis in older leaves	Insomnia, cardiovascular disease, immune dysfunction, type 2 diabetes mellitus, migrane, and many more	[35, 41, 100]
Sulfur, S	Inward puckered leaves, reduced shoot growth	Hyperhomocysteinemia, risk of cardiovascular diseases, and stroke	[65, 66, 100]
Iron, Fe	Interveinalchlorosis in younger leaves	Anemia, many infections, and inflammatory diseases	[2, 16]
Manganese, Mn	Interveinalchlorosis with a grey spot on leaves	Dermatitis, reduced clotting protein level, increased serum calcium and also associated with Down’s syndrome, Mseleni, epilepsy, and osteoporosis	[17, 130]
Boron, B	Chlorosis in young leaves where terminal bud become light green with shorter internode	Impaired brain functioning, bone health, and immune response	[120]
Zinc, Zn	Interveinalchlorosis and stunt growth. Kaira and white bud in rice and maize, respectively	The compromised immune system, retarded growth, and severe deficiency leads to Acrodermatitis enteropathica	[23, 121]
Copper, Cu	Chlorosis and necrosis in young tissue, male flower sterility	Dysregulation of lipid metabolism, anemia, myeloneuropathy, enteropathies inflammatory disease and affect the immune system too	[11, 158]
Molybdenum, Mo	Chlorotic mottling with necrotic spotting on leaves	Not observed	[124, 143]
Chlorine, Cl	Chlorotic molting and wilted foliage	–	[100, 160]
Nickel, Ni	Urea accumulation and necrosis in leaves	Reduced iron resorption leading to anemia, affected carbohydrate metabolism	[13, 84]

## Macronutrient elements

**Fig. 2:**
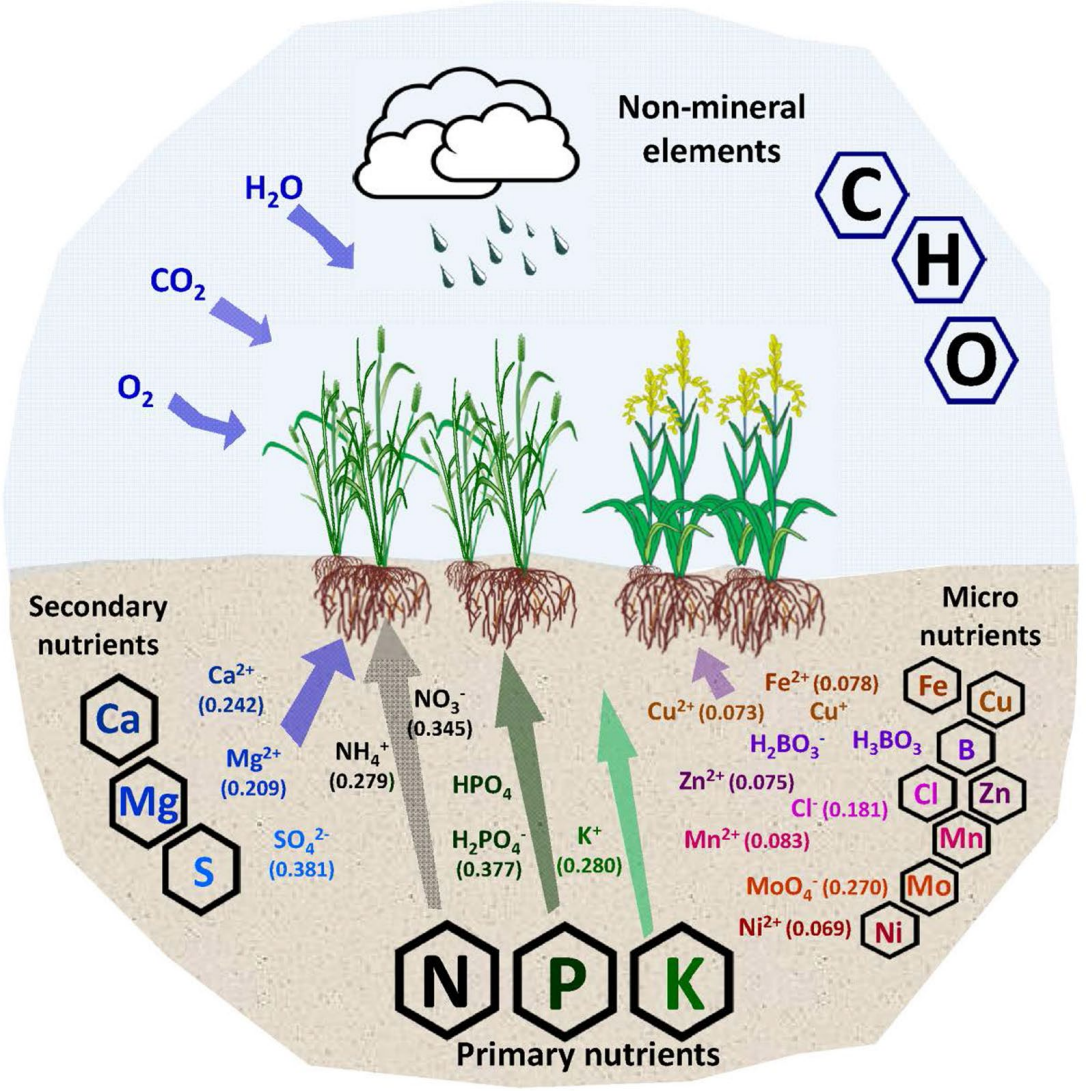
Available forms of essential nutrient elements for plant uptake and growth. Numbers in brackets indicate the hydrated radius (nm) of plant-available ionic species

These are the elements that are required by the plants in high concentrations. There is a total of six macronutrients (nitrogen, phosphorus, potassium, calcium, magnesium, and sulfur), and the most important element in the list is nitrogen (N). Theodore de Saussure in 1804 established N as the principal essential mineral element among all nutrients that play a key role in the development of the plant. Nitrogen constitutes around 1–5% of the total dry weight of the plant, and it is the essential constituent of the building blocks including amino acids, proteins, nucleic acids, enzymes, hormones, vitamins, secondary metabolites, etc. It plays a key role in photosynthesis as it is the main constituent of chlorophyll. N also governs the uptake and utilization of other essential elements. It promotes fruit development, improves the quality, imparts the green color to the plant, and improves vegetative growth [59, 60]. Although the earth’s atmosphere consists of 78% of inert nitrogen gas (N_2_), still it is deficient in agricultural soils and plants it cannot be directly absorbed [135]. It is absorbed by the plant roots in its inorganic form, and this is the only element, among all essential elements, that is absorbed both in cationic (NH_4_^+^, ammonium) and anionic (NO_3_^−^, nitrate) forms from the soil [22, 60, 112]. These NH_4_^+^ and NO_3_^−^ ions have a hydrated ionic radius of around 0.279 nm and 0.345 nm, respectively [100] (Fig. 2). These forms are absorbed by the plant through the specific transporter systems. Due to preferable concentration and mobility, NO_3_^−^ (1–5 mM) is the dominant source in plant absorption over NH_4_^+^ (20–200 µM). The total concentration of these inorganic forms of N present in the soil is only about 2% of the total N pool in soil, and the rest is organic N which constitutes about 98%, a form that is non-absorbable by the plants [59].

**Fig. 3:**
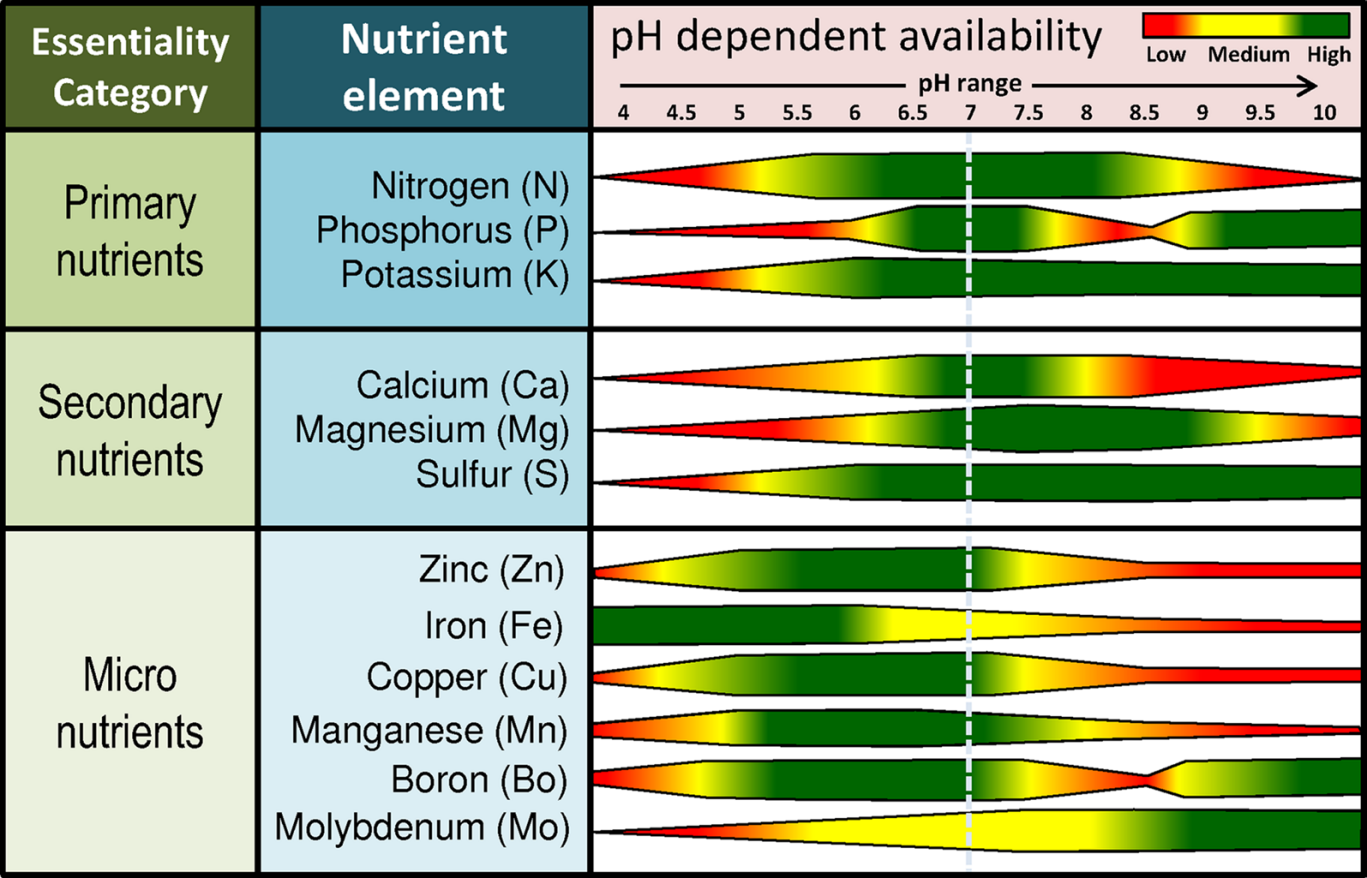
Essential plant nutrients and their availability based on the soil reaction (pH)

After N, Phosphorus (P) is the second most abundantly required nutrient for plant growth and is an important constituent of nucleic acids (DNA and RNA), phospholipids, and phosphor-proteins. Most importantly, it is the main component of the adenosine triphosphate (ATP), the key source of metabolic energy [57, 59]. In soil, P occupies the highest content, among other nutrients, in form of inorganic and organic P. Among these two, the inorganic P is the dominant form (54 to 84%) and is further categorized into three groups: solution P, labile P, and non-labile P based on the availability which is highly influenced by the pH of the soil. The primarily absorbable form of P by the plant is the anionic forms dihydrogen phosphate (H_2_PO_4_^−^) and hydrogen phosphate (HPO_4_^2−^) depending on the pH [60, 112]. The H_2_PO_4_^−^ has the crystal ionic and hydrated ionic radius of 0.377 nm [100]. The solution P, due to its high dissociation rate, is vulnerable to rapid conversion into labile P. This labile P consists of the bound or the P fixed to aluminum (Al), iron (Fe), and calcium (Ca) among which the Ca–P constitutes the highest proportion (40–50%). These forms are readily available in terms of quantity as compared to soil solution P but susceptible to cause P fixation where it becomes unavailable or non-absorbable by plants. The non-labile P is a less active fraction that is insoluble and unavailable. Further, the organic P comes from mineralization by the activities of microorganisms and phosphate [59, 135]. The best-suited pH for optimum availability of P is 6.5 to 7.5 (Fig. 3). As pH decreases, HPO_4_^−^ fixes with Fe and Al, and with an increase in pH, it gets fixed with Ca. Both these conditions lead to the formation of insoluble and unavailable compounds [109].

Potassium (K) is the 3rd most important among the primary nutrients and regulates several important metabolic activities like transport of water and nutrients, closing and opening of stomata, maintaining pH of the cytoplasm, and has a role in the activation of more than 60 enzymes. Potassium is known to enhance the defense mechanism of a plant. The K in the soil comes from the decomposition and disintegration of potassium mineral rocks and it is absorbed as K^+^ by the plants. It has 1–5% concentration in the healthy tissues, and it is a unique element that does not cause toxicity even at the high accumulation [59, 60, 119]. The K^+^ has the crystal ionic and hydrated ionic radius of 0.138 nm and 0.2798 nm, respectively [100]. The level of K in the soil increases with alkalinity or increase in pH of the soil and as pH decrease to acidic, it becomes less available (Fig. 3).

Calcium (Ca) is an essential structural and regulatory component that has an important role in the formation of the cell wall and calcium pectate that acts as a regulatory system for the entry of only non-toxic nutrients. It maintains the chromosomal structure, regulates the cell division, acts as cofactors for enzyme activation, and is essential for meristematic activities in the root tip [59]. This nutrient is absorbed by the plants in Ca^2+^ form, and it has the crystal ionic and hydrated ionic radius of 0.100 and 0.2422 nm, respectively [60, 100]. The availability of Ca increases with an increase in pH and decreases with acidity [109].

Magnesium (Mg) plays an important role in plants as it is the central constituent of chlorophyll. It exerts a crucial role in protein synthesis by activating the polypeptide chain synthesis that leads to amino acid formation, and it is also a structural component (bridging element) of ribosomes, which are considered as the protein factory of the cells. It also acts as a co-factor for several enzymes and promotes the uptake and translocation of other nutrients and macromolecules including phosphorous and sugar with the plant. Weathering of biotite, olivine, dolomite, epsomite, etc. is the source of Mg in the soil. Plants absorb this mineral as Mg^2+^ from the soil which is present either in exchangeable or water-soluble form. The Mg^2+^ has the crystal ionic and hydrated radius of 0.072 nm and 0.2090 nm, respectively [100]. As the soil pH decreases the Mg^2+^ ions tend to bind with Al^+3^ and result in Mg fixation. In acidic soils, around 65% to 70% of this Mg gets saturated and becomes unavailable to plants. Potassium, calcium, and magnesium are considered as the base cations, and their removal from soil leads to an increase in acidity [52, 109].

Sulfur (S) has an important role in the growth, functionality, and immune system of a plant where it provides strength to the plant to fight abiotic stress and biotic. It is a constituent of many enzymes, protoplast, and is also involved in the energetics of the plant cells. In soil, sulfur is present in both organic and inorganic forms where it is taken up by the plant predominantly in its inorganic SO_4_^2−^ form. SO_4_^2−^ is a highly mobile form that is transported through H^+^ mediated sulfate transporter. The transported SO_4_^2−^ is then stored in vacuoles and a part of this gets reduced to SO_3_^2−^, and finally to S^2−^ for the incorporation into cysteine and methionine amino acid. Although sulfur is highly mobile in the soil, in the plants it has only fair mobility due to which the newly growing tissues show deficiency symptoms under its reduced availability [98, 169]. The SO_4_^2−^ has the crystal ionic and hydrated ionic radius of 0.230 nm and 0.3815 nm, respectively [100]. A lower pH in the soil favors the availability of SO_4_^2−^ to plants and a higher concentration of SO_4_^2−^ in the soil further increases acidity in the soil due to the formation of sulfuric acid [109].

## Micronutrient elements

These are the essential elements that are required by the plant in low concentrations. The concentration and availability of micronutrients are highly affected by soil pH. The important nutrients in this category are iron (Fe), manganese (Mn), zinc (Zn), copper (Cu), boron (B), molybdenum (Mo), chlorine (Cl), and nickel (Ni). Iron is the 4th most essentially required nutrient by the plant to carry out the important metabolic activities including photosynthesis, cellular respiration, lipid metabolism, replication, gene regulation, electron transfer, and nitrogen fixation. It is an essential component of many proteins, enzymes, and also the structural and functional components of chloroplast, mitochondria, and vacuoles. Plants absorb this mineral as Fe^2+^ from the soil and due to its immobile nature, its deficiency first appears on younger leaves [141, 153]. The Fe^2+^ has the crystal ionic and hydrated radius of 0.072 nm and 0.078 nm, respectively. The concentration and availability of Fe increase with a decrease in pH of the soil [101]. Also, Fe is considered an acidic cation, and its higher concentration in the soil causes acidity [109].

Like iron, manganese (Mn) is also involved in several metabolic processes along with stress tolerance, photosynthesis, glycosylation, ROS scavenging, and ATP biosynthesis. Plants absorb this mineral as Mn^2+^ that acts as a cofactor for various enzymes including pyruvate carboxylase, superoxide dismutase, arginase, glutamine synthetase, and metalloenzyme cluster of OEC (oxygen-evolving complex) in photosystem II. Its deficiency generally shows in young tissue first due to its non-mobile behavior in the plant [8, 153]. The Mn^2+^ has the crystal ionic and hydrated radius of 0.080 nm and 0.083 nm, respectively. The concentration of Mn increases with decreased pH of the soil and becomes deficient at high or alkaline pH [33, 101].

The essentiality of Boron (B) for the growth of the plant cell was first recognized by Katherine Warrington in 1923, and later many investigators found it to be a critically essential micronutrient [153]. Plants take up B from the soil in the form of soluble H_3_BO_3_ and H_2_BO_3_^−^ [60]. It has an important role in sugar metabolism, flowering, fruiting, and seed development. Further, it also plays role in cell wall biosynthesis, lignification, fertility, and is necessary for the nitrogen cycle, with an important role in nitrogen fixation and nitrate assimilation. Boron plays a role in the defense system of a plant, and therefore has special importance, for commercial crops, e.g. for controlling rust in wheat (*Triticum aestivum*), and gray mold in grapes (*Vitis vinifera*) by inhibiting the spread of mycelia and germ tube elongation [55]. The concentration of B is found to be maximum at pH 6.0–6.5, and under alkaline conditions, the plant is unable to use B due to its fixation. At lower pH, the water solubility of B increases that also leads to its washing out or leaching [171].

Zinc (Zn) is an essential micronutrient required for the optimal physiological and biochemical functions of a plant. It regulates the defense system of the plant by protecting the lipids and proteins of the cell membrane from oxidative damage by playing role in the activation of antioxidant enzymes including superoxide dismutase, glutathione reductase, and peroxidase. Further, Zn has an important role in reproduction and grain development by regulating the remobilization of photo-assimilates [30, 31, 85]. Zinc is absorbed by the plant in the form of Zn^2+^ ions. It regulates the translocation of P as well as controls the toxicity caused due to excess P absorption in the plants. The Zn^2+^ has the crystal ionic and hydrated radius of 0.07 nm and 0.075 nm, respectively. The lower pH of soil favors the availability of Zn, and as we move to higher pH, the cationic Zn converts into anionic form, zincate ion, that forms a complex with calcium and reduces its availability [27].

Copper (Cu) is another very essential micro-nutrient for the growth of the plant as it regulates multiple metabolic and biochemical reactions including cell wall metabolism and signaling pathways during transcription. Copper further influences nitrogen metabolism, iron mobilization, protein trafficking, and has a role in the biogenesis of the molybdenum cofactor. It exerts a prominent role in photosynthesis, respiration, protein synthesis, protein tracking mechanism, phenol metabolism, regulates defense mechanism, enhances the fertility of male flowers, and acts as a stable cofactor for multiple enzymes. Cu is taken up by the plants from the soil in the form of Cu^2+^ ions. Cu is present in adequate amounts in most soils but its deficiency occurs due to its non-mobile behavior in the plant [153]. The Cu^2+^ has the crystal ionic and hydrated radius of 0.072 nm and 0.073 nm, respectively. The availability of Cu decreases with the increase in the pH of the soil [101, 109].

Molybdenum (Mo) is a crucial component of many proteins and enzymes specifically involved in nitrogen fixation including regulation of nitrogenase and nitrate reductase in legume plants. Apart from nitrogen fixation, Mo has an important role in regulating purine catabolism, amino acid (sulfur-containing) catabolism, ABA, Ureide, and Mocobiosynthesis. Molybdenum is present in the soil in several complex forms including ferrimolybdenite [Fe_2_(MoO_4_)], wulfenite (PbMoO4), and molybdenite (MoS_2_) but is absorbed by the plant in a water-soluble anionic (MoO_4_^−^) form. This form is also non-mobile within the plant, resulting in Mo deficiency in young tissues first [72, 115]. The MoO_4_^−^ has the crystal ionic and hydrated radius of 0.267 nm and 0.270 nm, respectively. The availability and uptake of Mo increase with the increased alkalinity [72].

Chlorine (Cl) is essential for the stabilization of membrane potential, electric excitability, and intracellular pH gradient attributed to its counter anionic (Cl^−^ fluxes) behavior. It plays a major role in maintaining cell turgor and osmoregulation. It is also involved in phosphorylation, photosynthesis reaction, and regulates the activity of many enzymes in the cytoplasm mainly enzymes involved in splitting water. It is highly mobile in the soil and is absorbed by the plant as a chloride ion (Cl^−^) [160]. The Cl^−^ has the crystal ionic and hydrated radius of 0.180 nm and 0.181 nm, respectively.

Nickel (Ni) has been reported for its essential function in many proteins, and as an activator of many enzymes, most importantly urease and Ni-urease for the metabolism of urea nitrogen. Ni has an important role in stress tolerance due to its ability to decrease the level of methyglyoxalase by regulating the GSH homeostasis and glyoxalase. It plays important role in ion metabolism too [42]. Nickel is absorbed in the form of Ni^2+^ from the soil, and owing to its high mobility it is easily absorbed in the higher amount by plants [14, 68]. The Ni^+2^ has the crystal ionic and hydrated radius of 0.067 nm and 0.069 nm, respectively. As the pH of the soil increases from 5 to 6.5, the uptake by plants and accumulation of Ni^2+^ increases [122].

## Shortfalls in achieving sustainable development goals via conventional fertilizers

The seventeen essential nutrients described in the earlier sections are naturally present in the soil environment but many times in their non-absorbable forms. The plant availability is also controlled by several factors like soil pH, soil moisture content, climatic conditions, nutrient harvesting, nutrient ion fixation, etc. Soil pH has significant control over the availability of a nutrient (Fig. 3). The nutrient fertilizers need to be applied in excess, to compensate for plant requirements as well as losses (leaching and volatilization) so as not to affect crop yield, nutritional quality, and fertility of the soil. Fertilizers played a very important role in the realization of the green revolution which resulted in a several-fold increase in crop productivity and yield, especially in Asia. These chemical fertilizers worked tremendously during the initial periods due to their immediate and predictable action, and reliability. But with time, the efficiency of conventional fertilizers has decreased, and consequently, the amount of fertilizer use increased to achieve the yield targets. This increased amount is further continuously increasing every year due to further decreased plant availability, and as a result, it has led to abusive use of fertilizers with severe negative impacts on the environment as well as human health. Most synthetic chemical fertilizers are made water-soluble with the purpose of ease of application and absorption of nutrients by the plant but due to this characteristic, the nutrients also tend to runoff with rainwater or leach down into the groundwater, and ultimately pollute lakes, rivers, and aquifers. Drinking water contaminated with these fertilizers can lead to several health problems. Nitrogen fertilizers release nitrate (NO_3_^−^) which is a highly water-soluble form of nitrogen. The nitrate may remain as it is for decades leading to methemoglobinemia in infants (blue baby syndrome), and other neurologic and endocrine defects. Drinking nitrate-contaminated water is even carcinogenic due to endogenous nitrosation that can cause cancer of the digestive tract [60, 112, 157]. These chemical fertilizers affect the natural beneficial flora and the organic material present in the soil by changing the physico-chemical properties, mainly the soil reaction. Ammonium sulfate and potassium fertilizers have been reported to increase soil acidity [133]. Continuous use of chemical fertilizers with only a few nutrient elements, as is a common practice by farmers, can degrade the soil structure and exhaust the essential nutrient base, especially for micro and trace nutrients [133]. Many a macronutrient fertilizers such as urea, which is a principal source of nitrogen in many parts of the world has a good water solubility and ready N availability to plants, but it also leads to loss of almost three-quarters of fertilizer nutrient during growth session, causing severe damage to the ecosystem [29]. Synthetic nitrogen fertilizers are the foremost contributors to nitrous oxide emissions and have been estimated to contribute as high as 20% to the world's total GHGs emissions [24, 111]. The nutrients are very essential for plant growth in a balanced concentration but an excessive or imbalanced application can also cause toxicity to plants directly or affect indirectly by encouraging pathogens. For example, the excess of nitrogen in comparison to phosphate makes crops susceptible to mosaic disease. Human exposure to chemical fertilizers can also be harmful with chances of skin and respiratory problems, if not handled properly. Ammonia as a chemical fertilizer has been reported to cause damage to lungs and eyes upon prolonged exposure [5].

## Purpose and benefits of moving up from conventional-ionic to nanoparticle feeding

**Fig. 4:**
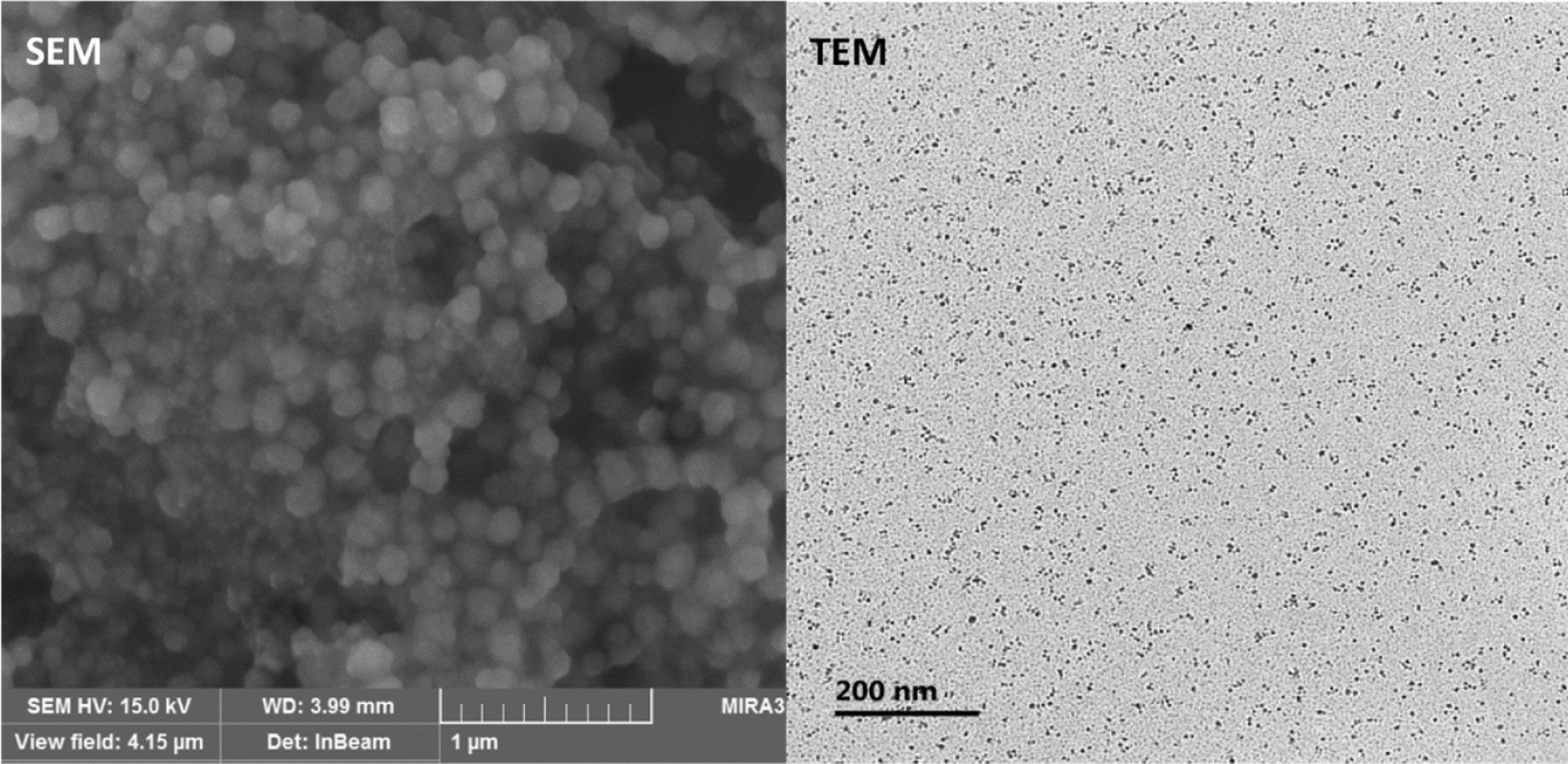
Micrographs of nanofertilizer particles as visible under a scanning electron (SEM), and a transmission electron microscope (TEM). Scanning electron micrograph shows CaSO_4_ nanoparticles, and transmission electron micrograph shows ZnSO_4_ nanoparticles (Source: Nanotechnology and Polymer Science Laboratory, CSSRI, Karnal, India)

**Fig. 5:**
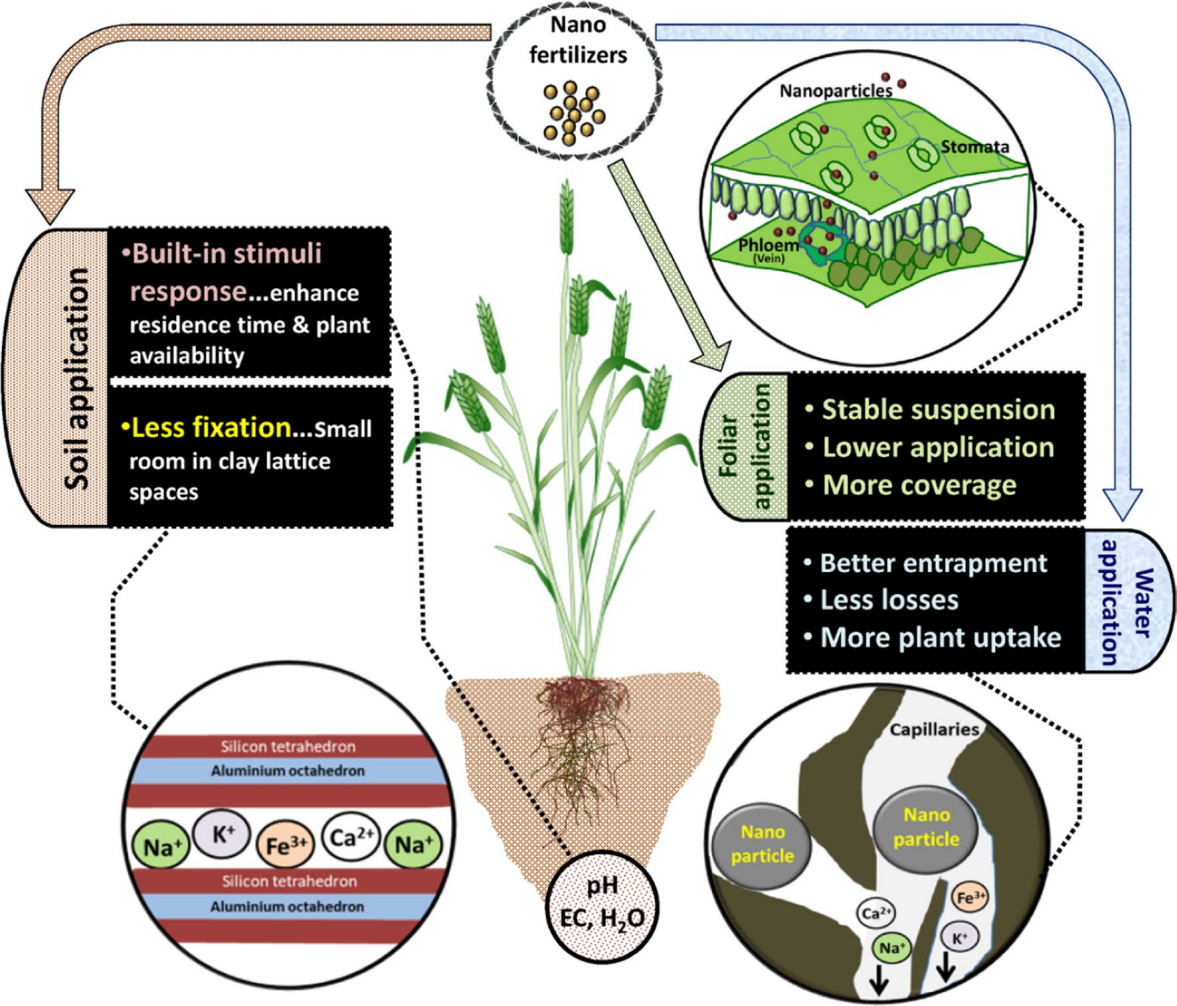
Potential benefits of nanofertilizers under different modes of application for crop production

Fertilizer application to the crops in the form of nanofertilizers is gaining immense attention, worldwide. Ions have the size approximately in angstrom (10^−10^ m) to picometer range (10^–12^ m) whereas nano-particles have their size in the nanometer range (10^–9^ m) (Fig. 4). The lattice diameter of clay has the size in angstrom (Å) where nutrient ions in the picometer size get fixed in the inter-spaces [100]. Let’s take an example of the ammonium ion (NH_4_^+^) that has a diameter of 2.8 Å which is close to the inner lattice space diameter of 2.89 Å. Due to this diameter similarity, the ammonium ions get fixed into the clay lattice and become unavailable to the plants and the plant beneficial microbes. Further, ions are rapidly leached out from the soil and contaminate ground water, as in the case of nitrate ions. NPs are 10 to 100 times bigger in size than ions (Fig. 2), and therefore they have lesser chances of fixation in the clay lattice spaces, and therefore are less likely to be leaching out from the soil system compared to ions. Further, the nanofertilizers with NPs embedded in a matrix can be considered as a kind of lunchbox system for the plants where plants can have their reserve nutrient food and use it when conditions become optimum for plant uptake. Stimulus responsive polymer matrix can also provide enhanced release of nutrients only when conditions are optimum (e.g. moisture, pH), and therefore they would even be climate-smart (Fig. 5). The essential nutrients like Mo, Cu, Mn, and Fe have immobile nature in soil but are fairly mobile once they enter into the plant system [8, 115, 153]. Providing such nutrients a polymeric carrier in nanometer size can ease their transport via the free passage and through molecular transporters, and provide a better option for their fortification in plants to overcome their deficiency. There can be several purposes of formulating nano-fertilizers as discussed in the following sections.

## Controlled release of nutrients

Controlled release of nutrients in the soil is needed to minimize the losses (via leaching and runoff), provide stability, and prolong the availability of the nutrients. An ample amount of work has been conducted and reported for the controlled release of nutrients. Mohanraj et al*.* [110] performed their study on rice (*Oryza sativa*) for controlled release of NO_3_^−^ and NH_4_^+^ from nano-fertilizer and they found an increase in the duration of release, from 12 to 20 days, in comparison to conventional ammonium sulfate. The release kinetic of nutrients from chitosan-NPK nanofertilizer applied to coffee (*Coffea* sp.) plant was tested by immersing the synthesized nanofertilizer in a solution of pH 5.5 for 240 h, and nutrient concentration was analyzed at successive 24 h intervals [56]. Up to 48 h, a very slow release of 13% of nitrogen was observed that increased up to 60% after 72 h, and remain constant up to 192 h with only a slight increase to 66.7%. Potassium release kinetic showed a release of 55.4% in the first 72 h that become constant at 58% for next 240 h. Phosphorous represented the slowest release kinetic of 3% throughout the examination period. A hybrid nanofertilizer (HNF) formulation was prepared where nanourea-modified hydroxyapatite NPs were generated for sustainable and slow release of nitrogen, calcium, and phosphate into the soil [150]. Here the synthesized nanofertilizer was prepared from available conventional fertilizers where firstly, the hydroxyapatite NPs were prepared which were then modified with synthesized nanourea and finally mixed with chemically synthesized Cu, Fe, and Zn NPs to obtain the HNF. The nutrient release study of HNF was performed on *Abelmoschus esculentus* (Ladies finger) via both normal tap water and soil water for 14 days. The results revealed a sustained release for 7 days and a sequential increase of Ca^2+^, NO^2−^, NO^3−^, Cu^2+^, PO_4_^3−^, Fe^2+^, and Zn^2+^ up to 14 days that led to enhanced use-efficiency and increased yield as compared to the conventional fertilizers.

## Stimuli-response and site-specific release

To avoid the photolysis, volatilization, and degradation, as noticed in the case of conventional fertilizers, stimuli-responsive nanofertilizers offer a better way by providing site-specific, controlled, and smart release of nutrients. Generally, these stimuli include response to change in pH, photo or thermal conditions, enzymatic, and redox reaction. Zhang et al. [166] performed a study based on an anion-responsive nanofertilizer composed of polyethylenimine-modified hollow mesoporous carbon NPs for the delivery of selenium loaded via electrostatic interaction. In this nanoformulation the concentration and valence of anions including, PO_4_^3−^, CO_3_^2−^ and OH^−^ triggered the release of selenium in a controlled or target-specific manner. The results revealed a 60% increase in plant growth as compared to the control due to the enhanced selenium utilization efficiency, without reduced losses. Further, a redox responsive nanoformulation was developed by Sun et al. [148] for the delivery of abscisic acid in *Arabidopsis thaliana* plant under hydric conditions. The release of abscisic acid was triggered in response to the intracellular glutathione in this mesoporous silica nanoformulation.

## Dosage reduction

By using nanofertilizers instead of their conventional counterparts, the amount of required fertilizer for crop production can be reduced by a significant amount. This reduction in the required fertilizer dose has multiple benefits as it will reduce the negative impacts of fertilizers on the environment, and would make it cost-effective too. A study by Aziz et al*.* reported that the reduced amount of a nanofertilizer, 10% chitosan-NPK fertilizer, was able to give a significant increase in crop index, harvest index, and mobilization index as compared to the conventional fertilizer in wheat crop [18]. Similarly, Ramirez-Rodriguez et al*.* [136, 137] reported using urea-doped calcium phosphate for the *Triticum durum* plant to reduce the required dosage of nitrogen. Their results revealed that by reducing the N dosage to 40% in the form of nanofertilizer in comparison to the maximum dose of conventional fertilizer via foliar and root application, the growth and yield were unaltered which meant that nanofertilizer with 40% reduced amount performed better than the conventional fertilizer. They also observed that uptake of these fertilizers via root application was quite faster (within 1 h) as compared to the foliar application which takes 2 days. Recently, Tarafdar et al*.* [150] also found that nanourea-modified hydroxyapatite nanoformulation resulted in a reduced requirement dose of 50 mg per week as compared to the conventional fertilizer dose of 5 g per week.

## Minimize environmental losses

The runoff and leaching of mineral nutrients into the water bodies (surface and subsurface aquifers) results in their significant accumulation with time and results in pollution of drinking water. The enrichment of surface water bodies with lost plant nutrients also results in algal bloom and eutrophication. Phosphate fertilizer overuse is the main contributor to eutrophication [60, 112]. Phosphorus-based nanofertilizers hold a huge promise for improving water quality and in checking water pollution, worldwide. Liu et al*. *[179] performed a comparative study with a conventional phosphate fertilizer and synthesized carboxymethyl cellulose stabilized hydroxyapatite nanoformulation on soybean plants in a greenhouse experiment with an inert growing medium. The formulation provided sufficient P to the crop and had less mobility in the environment which in turn resulted in less availability to the algae, and therefore it can be an important alternative to convention P fertilizer [91].

Agriculture accounts for 11% of the global anthropogenic emission of greenhouse gases (GHGs) mainly attributed to the manufacturing of synthetic fertilizers, specifically nitrogen fertilizers, and the use of fertilizers during crop production. Energy use and GHG emissions (CO_2_ eq.) in fertilizer production, transportation of fertilizers and loss in the environment in gaseous (N_2_O, NH_3_) or aqueous form (NO_3_^−^–N) is a significant percentage of the total contributions to global warming by all agricultural activities together. So, fertilizer management to minimize losses and improve use efficiency can be a promising strategy to surmount the issue of global climate change. In this direction, nanofertilizers have been reported to provide a better alternative [24, 111]. Mohanraj et al. [110, 111] reported two studies with a significant decrease in GHG emissions in rice soil with the use of nano zeolite-based nitrogenous fertilizer containing NO_3_^−^ and NH_4_^+^ forms of N. The recorded size of the two forms was 66 nm and 77 nm for NO_3_^−^ and NH_4_^+^, respectively, with an irregular shape. The result of GHG measurements showed that the N_2_O emission decreased to 0.88 mg m^−2^ day^−1^ with nanofertilizers in comparison to 1.67 mg m^−2^ day^−1^ with conventional ammonium sulfate. Further, CH_4_ efflux also decreased significantly with the nano-zeolite fertilizer use. A 50% decrease in the overall emission of GHGs was reported due to nanofertilizer use.

## Better efficiency under stresses

Salinity stress is one of the major concerns in crop production and it affects an estimated 20% of the cultivated land, and 30% of irrigated agricultural land, globally. Saline conditions can impose different types of limitations for economical crop production including restricted nutrient availability to plants, osmotic stresses, and physical barriers to root growth [127]. Siddiqui et al*.* [146] formulated Nano-SiO_2_, and they reported it to be beneficial for crop production under saline soil conditions. The application of this nanofertilizer to tomato (*Lycopersicum esculentum*) plants resulted in increased plant growth, chlorophyll content, protein content, and seed germination along with the increased dry and fresh weight of crop. A comparative effect of a conventional and a nanoformulation of iron sulfate was tested on sunflower (*Helianthus annus*) grown under two saline conditions, with salinity levels of 0 and 100 mM NaCl concentration [152]. The treatment was done via foliar application in both cases, and results revealed that the chlorophyll content increased from 36% under conventional fertilization to 57% with nanoformulation application. Biomass and growth also increased with the use of nanoformulation. Yet, at 100 mM, the extent of increase in these parameters was not very high but significantly as compared to conventional fertilizer. Zinc nanofertilizer treatment in the form of ZnO NPs has also been reported to improve plant characteristics including plant height, tillers, spike length, and grain yield in the wheat plant under saline conditions [132]. Nanofertilizers may play a crucial role in saline and hyper-saline environments. Membrane permeability to nutrients in nanofertilizers is more than conventional fertilizers, so the nutrients can easily enter the plant [172]. Therefore, in saline conditions where the plant is exposed to osmotic stress due to salts in the soil solution, the cells need less water for absorbing nutrients, in the case of nanofertilizers. Less water intake for nutrients has its other advantages such as lesser intake of harmful ions like Na^+^ into the plant system or decrease in the water requirement by the plant. Salinity-prone areas often have salt-laden ground-waters, and lesser use of such waters for crop irrigation helps in lessening the problem of secondary salinization. Nanofertilizers provide benefits for use in marginal soils and with the use of salt-laden waters.

Drought stress is another major concern in crop production. Nutrient management is critical under drought conditions as transport of nutrients takes place via water in the soil. Nanofertilizers have come up as an important alternative strategy to cope up with drought stress. Among nanoformulations, metal-based nanoformulations have performed great in this area [34, 90]. Nano-ZnO treatment improved the drought tolerance of the maize (*Zea mays*) plants by promoting the endogenous synthesis of melatonin which inhibits the accumulation of malondialdehyde and osmolytes in the leaves [149]. It also resulted in the activation of antioxidant enzymes which in turn control the drought-induced damage to the organelles including chloroplast and mitochondria. Furthermore, an ample amount of work has been reported for Nano-TiO_2_ and Nano-SiO_2_ under drought-stress conditions [4, 15, 79]. Nanourea-modified hydroxyapatite nanoformulations have been reported to reduce fertilizer requirement as compared to conventional fertilizers, and their use also increased the water holding capacity of the soil up to 80%. This increase in water holding capacity was attributed to the high porosity of the nanoformulation, and the prolonged observations found the soil to retain this water holding capacity [150].

## Categories of nanofertilizers

Nanofertilizers can be categorized into three broad groups based on their constituents or based on the strategy used for their preparation (involving both top-down and bottom-up approaches). These three categories do not have clear and specific boundaries, and they may be considerably overlapping, and may also involve as well as a combination of more than one principle [107].

## Nanoscale input fertilizers

This category involves the reformation of already existing fertilizers at the nanoscale dimension either alone or in combination with other constituents in the form of emulsions and particles to reduce the size of the active ingredients. This category comes under the top-down approach where nanoformulations are prepared via sizing down the bulk material. This can be achieved via both chemical and physical methods. Nano-urea, nano-ammonium salt (chemical), nano-peat, and nano-bacterial compositions (organic) are the representative fertilizers of this category. The green approach of synthesis of NPs from the plant extracts and microorganism-based extracts also comes under this category. A nano-emulsion with neem cake and plant growth-promoting rhizobacteria is an example of this nanoscale fertilizer input category which was designed to obtain the slow release of nutrients and growth hormones simultaneously [99]. The nanoformulation was tested on the green gram (*Vigna radiata*), and its application resulted in increased grain weight, protein concentration, and yield. Further, a highly biodegradable and biocompatible nano-urea-NPK (nanoU-NPK) was synthesized via a green method for slow and gradual release [136, 137]. This nanoformulation acted as a multi-nutrient fertilizer because of being a combination of constituents including calcium phosphate NPs doped with nitrogen, phosphorous, and functionalized with urea. This nanoU-NPK was tested on durum wheat where it resulted in a 40% reduction in the amount of nitrogen required as compared to the conventional urea treatment.

Another recent study reported an HNF formulation wherein nanourea-modified hydroxyapatite NPs were formulated for the slow release of nitrogen, calcium, and phosphorus into the soil [150]. The synthesized nanofertilizer was prepared from available conventional fertilizers, where firstly the hydroxyapatite NPs were prepared which were then modified with synthesized nanourea and finally mixed with chemically synthesized Cu, Fe, and Zn NPs to obtain the HNF. The obtained particle sizes were 39.76 nm and 38.21 nm for nanourea and hydroxyapatite, respectively, as determined with SEM. Further, the successful intra-particle bonding and interaction were evaluated via XRD analysis. The studies on the efficacy of HNF were performed on Okra (*Abelmoschus esculentus*) via soil treatment, and the results revealed a sustained release with a sequential increase in Ca^2+^, NO^2−^, NO^3−^, Cu^2+^, PO_4_^3−^, Fe^2+^, and Zn^2+^. The HNF maximized nutrient use efficiency and improved yield as compared to conventional fertilizer.

## Nanoscale additives fertilizers

As the name indicates, this category involves the addition of a nanoscale material or formulation to existing macroscale traditional fertilizers [107]. The addition of nanoscale materials is not intended to improve the nutritional performance of the fertilizer, they are rather added to provide additional advantages like improved water absorption, water retention, water transport, cell wall extension, and soil stabilization [80].

In this category, carbon nanomaterials including carbon nanotubes (CNTs) and carbon nanofibers (CNFs) are the most studied and explored for their potential in improving productivity. CNTs are categorized as single-walled carbon nano tubes (SWCNTs) and multi-walled carbon nanotubes (MWCNTs). CNTs are reported to increase the water uptake in plants either by increasing the molecular transport via xylem or via decreasing the porosity in coarse-textured soils, and ultimately reduce loss of water. Martinez-Ballesta et al*.* [102] used MWCNTs for enhancing the water uptake along with improved growth in broccoli under salt-stressed soil conditions. They hypothesized that the CNTs caused physical changes in cells and tissues of the roots resulting in more Na^+^ retention that led to increase uptake of water by roots.

A comparative study between CNTs and CNFs was performed for the stabilization of a clayey-sand soil in terms of optimizing water content, hydraulic conductivity, specific gravity, pH, and dry density [10]. The results of the study indicated that both nanomaterials resulted in significantly different outcomes for hydraulic conductivity, which was significantly reduced by CNFs compared to CNTs.

## Nanoscale host fertilizer

This category involves fertilizers or nutrient supplements that are adsorbed, entrapped, or encapsulated into the nano-pockets, nano-pores, nano-films, or within any type of nano-spaces of the host materials (Table 2). This category involves a combination of both physical and chemical methods of synthesis. An ample amount of work has been done in this category of nanofertilizers and reports confirm that the coated or encapsulated nanomaterials are rather better as compared to non-coated nanoparticles in terms of stability, slow release of nutrients, and bio-safety as well [27]. A study done by Abdel-Aziz et al. [18] is an example of nanoscale host fertilizer where they used chitosan (CS) and polymethacrylic acid (PMAA) NPs (CS-PMAA) as host for loading of nitrogen, phosphorous, and potassium for the foliar application in the wheat plant to enhance the growth as well as productivity in sandy soils. They performed a comparative analysis for two seasons under the outdoor conditions for foliar application of different concentrations (10%, 25%, and 100%) of both conventional NPK fertilizer, and CS-PMAA-NPK synthesized via solution dropping method. The results indicated a significant increase in all the measured variables, including the number of spikelets, grains per main-spike, spike length, shoot length, plant height, etc. in the treatment, compared to control. Comparatively higher effects were achieved with CS-PMAA-NPK 10% treatment where the plants reached their harvesting stage by 23.5% shortened life cycle period (130 days as compared to 170 days) in comparison to control and conventional NPK fertilizer. The uptake and translocation of NPs occurred through phloem tissues which were confirmed via TEM analysis.

**Table 2:** Characteristics and use of some nano-scale host fertilizer formulations for plant nutritive needs

Nanoformulation	Host	Characteristic	Aim	Test plant	Treatment	Ref.
Nano-CS-NPK	CS + PMAA	No data	To enhance growth productivity in sandy soil	Wheat (*Triticum aestivum*)	Foliar application with 3 times at 3-weeks intervals In outdoor condition for 2 seasons	[18]
Nano-NPK	CS + PMAA	Uncontrolled morphology with the actual particle size of 8–9 nm by TEM	To check the effect on fruit yield and quality	Cucumber (*Cucumis sativus*)	Irrigation in a randomized complete block design with 3 replicates	[106]
Zinc nanocarrier	CS + TPP	H.D: 325 nm, ZP: + 42.34, spherical morphology, and actual particle size 200 nm by SEM	Agronomic fortification in grains	Wheat	Foliar application twice a week for 5 weeks	[36]
Selenium nanofertilizer	PEI- carbon	100 nm with spherical shape	Controlled and site-specific delivery	10 different vegetable plants	Soil amendment	[166]
Urea-hydroxyapatite nanofertilizer	Hydroxyapatite	Crystalline size of 18 nm with bead-shape	Slow-release of nitrogen	Rice (*Oryza sativa*)	Soil amendment	[82]
Zinc CS nanoparticles	CS + TPP	H.D: 387 nm, ZP: + 34 mV, spherical morphology, and actual particle size in 200–300 nm by TEM	To promote the crop yield with disease control	Maize (*Zea mays*)	Seed priming 4 h before showing and foliar applicationwhere spray of nanoformulation was done after 35 days of sowing	[31]
Chitosan-PMAA-NPK nanofertilizer	CS + PMAA	The actual particle size of 38.98, 87.65, and 24.07 nm by TEM, for N, P, and K respectively	Dose-dependent genotoxic and mitotis effect	Pea (*Pisum sativum*)	Root applicationon 5-day-old seedlings for 1, 2, 4, and 7 days	[78]
Zn/B nanofertilizer	CS + TPP	H.D: 700 nm, ZP: + 35 mV	Biophysical characteristic and growth effect on seedlings	Coffee (*Coffea* sp.)	Foliar application 3 times in 20 days in the greenhouse	[156]
Zn nanofertilizer	Nano-zeolite	Cubic to round shape	Slow and controlled release of Zn	–	–	[164]
NPK-nanofertilizer	CS + TPP	H.D: 500 nm, ZP: + 50 mV, spherical morphology	Biophysical characteristic and growth effect on seedlings	Coffee	Foliar application 3 times in 15 days in the greenhouse	[56]
Nano Zn fertilizer	CS + TPP + Zein protein coating	H.D: 709 nm, ZP: + 36 mV, spherical morphology, and actual particle size of 80–300 nm by TEM	Slow and controlled released	Cotton (*Gossypium hirsutum*)	Foliar application 25 mL of 50 ppm concentration spray on 30 days old plant	[73]
Potassium nanofertilizer	CS + alginate	H.D: 594 nm, and actual particle size of 151 nm by SEM	Maximum loading with controlled release of potassium	–	–	[119]
CNK fertilizers	CS + MAA	ZP: + 21.8 mV, spherical morphology and actual particle size of 39–79 by TEM	Soil conditioning with biomass production	Maize	Randomized completely block design (RCBD) and consisted of eight treatments in an open field	[83]
Sulfate-supplemented NPK nanofertilizer	CS + TPP	H.D: 145.4 nm and 450.5 nm with ZP = 14.7 mV and 19.9 mV, respectively	Effective growth of plant	Maize	Soil treatment under greenhouse condition	[37]
Chitosan (Cu and SA) nanofertilizer	CS + TPP	H.D: 539.7 nm, ZP: + 37.3 mV, spherical morphology	To improve the source activity of the plant	Maize	Seed treatment 4 h before showing and foliar application sprayed after 55 days of seed showing	[144]
Urea-doped calcium phosphate nanoparticles	Ca-P	Actual particle size-13.8 nm, with disk-shaped morphology	To reduce nitrogen dose requirement	Durum wheat (*Triticum durum*)	Foliar and root application	[136, 137]

Another similar study on testing of nano NPK ingredients encapsulated in CS and PMAA was performed on cucumber (*Cucumis sativus*) plants to evaluate the effect of fertilizer on growth, yield, and quality under greenhouse conditions [106]. The nano-particles were prepared via a top-down chemical synthesis approach under 2 MPa pressure. The comparison of nanoformulations was done to conventional fertilizers and untreated control, and results revealed an increase in yield to an extent of 4.84% and 53.42% in the first and second seasons, respectively, along with improved quality and other plant growth parameters. This study also revealed that the interaction and response of the nanoformulations did not follow the same pattern, and it varied with species, type of nanoformulation, and growth stage at the time of treatment. Deshpande et al. [36] synthesized zinc-loaded chitosan/TPP NPs where the intermolecular linkage between positively charged chitosan and negatively charged sodium tri-polyphosphate (TPP) led to the formation of mesh or pockets for loading of ZnSO_4_. The nanoformulation was prepared with the objective of agronomic bio-fortification of Zn in wheat via foliar application. The Zn translocation into leaves and seeds was evaluated using Zinquin and dithizone, respectively, due to the good binding efficiency of these two with Zn. The microscopic images revealed that zinquin exerted an enhanced fluorescence in leaves from where it was transported to the sink organ. The staining results revealed that dithizone enhanced reddish-pink color in the endosperm with nanoformulation treatment, as compared to the control. The results from quantitative analysis indicated an increase in grain Zn content by 27% to 42% in different varieties. This enhanced Zn in grains represented a successful nutrient fortification in wheat.

In another study, ZnSO_4_ encapsulated in chitosan NPs was tested to promote crop yield and disease control in maize [31]. 1 g of NPs contained 33.47 mg of Zn^2+^, representing 82% entrapment efficiency. The release of NPs was reported to exert slow and sustained release efficiency with an increase in pH from 1 to 7. The nanoformulation showed significant antifungal activity against *Curvularia lunata*, which is responsible for around 60% of yield loss via *Curvularia* leaf disease. The formulation was also noticed to strengthen the innate immune system of the plant via enhancing lignin content, antioxidants, defense enzymes, and by influencing ROS balancing. The grain yield and the zinc content were increased by 39.8% and 62.21 µg/g dry weight, respectively. In yet another case, Kanjana [73] worked on a nano-zinc fertilizer where Zn was loaded into TPP-crosslinked-chitosan NPs, and coated with zein protein. The size of the particles was obtained in the range of 70–86 nm and 80–300 nm for uncoated and coated NPs, respectively. The zein coating on these particles resulted in increased Zn entrapment efficiency but decreased loading efficiency as the size of the particles increased with increased coating percentage. The effect of these NPs was evaluated in pot experiments with cotton (*Gossypium hirsutum*) plants via foliar spray with 50 ppm Zn concentration. The application of nanoformulation resulted in increased root length, a higher number of leaves, and increased plant height within 7 weeks as compared to non-coated NPs. So in all, their results indicated the coating of zein protein further improved the effect of nanoformulations for slow and controlled release of Zn.

Sharma et al. [144] synthesized a host nanoscale fertilizer for co-encapsulation of CuSO_4_ and salicylic acid (SA) for foliar application and seed treatment in maize to get the enhancement of its source activity via the slow and sustained release of nutrients for an extended period. The synthesized particles were in a monodisperse stable form with a PDI (polydispersity index) and zeta potential of 0.13 and + 37.3 mV, respectively. The synthesis was done via the ionic gelation method where the entrapment efficiency of NPs was obtained to be near 60% and 55%, loading concentration of 35% and 26%, and release efficiency of 64% and 35% for Cu and salicylic acid, respectively. The treatment results in maize were compared with the untreated control, and the enhanced activity of α-amylase (1.7-fold), protease enzyme (threefold), and increased SVI (seeding vigor index) (1.5-fold) was obtained with the nanoformulation. The antioxidants activity, chlorophyll content, and sugar translocation activity also increased after 24 h of treatment of nanoformulation, due to the synergic effect of Cu and SA.

## Potential roadblocks in the use of nanofertilizers

Nanofertilizers have perceivable advantages yet nanofertilizers have not been able to make significant inroads into industrial-level production or real farming-world use. Most studies on nanofertilizers development and use show benefits in terms of enhanced nutrient uptake by the plants, increased crop yields, and decreased losses to the environment [21, 70]. Yet, several issues hinder the growth of nanofertilizer industry and subsequent use of nanofertilizers in the real world, at least for the time being. One of the major concerns with nanoscale materials, in general, is the toxicity caused to the plants, microbes and animals [26]. The potential toxicity related concerns are discussed in the following sections.

## Nanotoxicity and genotoxicity issues

Concerns have been raised regarding the toxicity effects of nano forms, especially metal and metal oxide NPs. Among nanoformulations, metal and metal oxide-based NPs have been studied extensively and reported to exhibit growth promotion and improved source activity in crop plants. Apart from their promising advantage in increasing nutrient uptake and efficiency, metal oxide NPs have also been reported to exert nanotoxicity and genotoxicity related impacts in crop plants. A study revealed that the most commonly used metal oxide NPs such as CuO, CeO_2_, TiO_2_, Ag, NiO, and ZnO can exert nanotoxicity via oxidative stress and molecular-level damage [96, 105, 108]. Fullerenes and single-walled carbon nanotubes are some non-metal NPs that have also been reported to exert nanotoxicity [61, 138]. There are very limited studies on genotoxicity in crop plants but it could also be the most adverse impact of nano-particles which is now gaining the attention of the scientific community [20, 74]. It is reported that the genotoxicity of NPs is inversely proportional to the size and directly proportional to concentration and time of exposure as these factors favor the accumulation of NPs in plants. The toxicity seems to increase with a decrease in size [25]. Xiang et al. [161] noted the effects of both particle size and morphology on toxicity effects. In this study, large columnar ZnO-90 and small spherical ZnO-50 had similar toxicities. Most toxicity-related studies are confined to sizes less than 100 nm and therefore, size effects above this range could not be ascertained. Smaller size NPs, approximately 8–10 nm, can easily cross via nuclear pores and have direct access to the genetic material. On the other hand, larger NPs can only have direct access to DNA during cell division. Further, many more studies supported these findings [97, 140, 155]. Genotoxicity mechanisms of NPs can be categorized into two groups: Direct genotoxicity (where NPs directly damage the DNA either mechanically or via chemical bonding), and indirect genotoxicity (which includes, ROS generation, reduced DNA repair, and interaction with nuclear protein) [74]. The mechanisms involved in nanotoxicity and genotoxicity are as follow:

## DNA damage

Micronuclei formation, chromosome fragmentation and disturbance, stickiness and bridging of chromosomes, decreased mitotic index, and laggard’s chromosomes are some of the prominent anomalies noted in plants exposed to some metal oxide NPs and carbon nanotubes [74]. DNA damage via either direct or indirect mechanisms can be the most devastating effect of NPs on plants, and humans. Metals and their oxides have been reported to exert direct genotoxicity. The work of Faisal et al. [44] using Nickle oxide (NiO) NPs indicated direct genotoxicity in the tomato plant where these NPs were able to directly access the DNA and caused irreversible damage in cells. Another study with cobalt oxide NPs (Co_3_O_4_ NPs) reported indirect DNA damage in eggplant that lead to apoptosis in plant cells [43]. The DNA damage occurs as a result of degeneration of mitochondrial cristae, peroxisome proliferation, NO generation, and vacuolization. The mitochondrial damage was analyzed by fluorescence of mitochondrial specific dye Rh123 that leaks out in the cytoplasm as a result of swelling in mitochondria.

Even at very low concentrations of 5, 10, and 20 mg, biogenic Ag NPs from *Swertia chirata* have been reported to exert chromosomal aberration in both mitotic and meiotic cells in root tip and flower buds of *Allium cepa* [142]. Ghosh et al. [49] also noted loss of membrane integrity, DNA strand breakage, and chromosomal damage with the use of ZnO nanoparticles in *Allium cepa*, *Nicotiana tabacum* and *Vicia faba.* Akhavan et al. [6] noted genotoxic effects on human mesenchymal stem cells through DNA fragmentation and chromosomal damage even at concentration of 0.1 µg mL^−1^ for reduced graphene oxide nanoplatelets. Lovecka et al. [95] performed tests for size-dependent genotoxicity effects of silver and gold NPs in the *Nicotiana tabacum* L. plant. The study revealed that the 12–25 nm of silver nanoparticles (Ag NPs) exert more damage to DNA in leaves as compared to the Ag NPs having a size range of 22–25 nm. Similar results were observed with gold nanoparticles (Au NPs). Further, their study also revealed comparative toxicity of Ag NPs and Au NPs where the toxicity exhibited by 30 mg kg^−1^ Ag NPs was reported to the similar to 100 mg kg^−1^ of Au NPs indicating that Ag NPs are more toxic for the plant as compared to the Au NPs.

## Cell wall membrane damage, physical trapping, and wrapping of cells and tissue

Another nanotoxicity mechanism is the cell wall membrane damage where the sharp edges of NPs are responsible for damaging cell walls, resulting in the leakage of cellular content in the surroundings and ultimately resulting in cell death [7]. Mazumdar and Ahmed [104] reported a study of Ag NPs toxicity in *Oryza sativa* where the results of TEM image revealed that the particles ruptured the cell wall and damaged the vacuoles of root cells. At lower concentrations (up to 30 µg/mL), no damage was observed but at higher concentrations, the cell wall was heavily damaged. The study indicated that this mechanism of nanotoxicity is directly proportional to the concentration of NPs.

Karlsson et al. [76] performed a study with Cu metal, Cu–Zn alloy, and CuO NPs, and revealed that the NPs release their metal at the surface of the cell membrane where CuO was responsible for the generation of reactive oxygen species. Nhan et al. [118] presented the TEM image of approximately 10 nm-sized CeO_2_ NPs in the leaves of the cotton plant reported to damage the chloroplast membrane along with vascular bundle fragmentation. A recent study by He et al. [61] showed fullerene NPs (nC60) blocking the pores of root cells. The electron microscopic images revealed the squeezed condition of endothelial cells and also extensive damage in the inner wall of root cells. Further, a long-term exposure resulted in complete blockage and altered membrane fluidity under this study. Silica NPs were used for studying the wrapping mechanism of cells where small silica NPs of 18 nm were reported to exert a freeze effect on the fluid phospholipid [167]. On the other hand, larger silica NPs caused wrapping of the membrane resulting in an excessive increase in lipid lateral mobility that eventually led to the collapse of vesicles. Hashemi et al. [58] noted physical trapping of spermatozoa by graphene oxide nanosheets resulting in the inactivation and death.

## Oxidizing through ROS generation, and nanobubbles

Reactive oxygen species (ROS) mediated stress is one of the main mechanisms of nano-toxicity that can instigate damage either via physical contact or through release of toxic ions after dissolution of nanoparticles [1]. Four types of ROS were identified by Liu et al. [93]: Super oxide anion radical (O_2_^**.**−^), hydrogen peroxide (H_2_O_2_), hydroxyl radical (OH·), and singlet oxygen (^1^O_2_). Faisal et al. [44] studied NiO–NPs toxicity in *Solanum lycopersicum* where these particles exerted toxicity via excessive ROS generation. This oxidative stress triggered the mitochondria to release caspase 9 and helped generating NO that jointly favored the DNA damage and ultimately led to apoptosis in root cells. The toxicity of such metal oxide NPs via oxidative stress has been noted to be a dose-dependent mechanism where the level of hydrogen peroxide, lipid peroxide, and other reactive oxygen species increased with increasing concentration [71, 145].

Mukherjee et al. [114] performed a study in green pea that indicated that the ZnO-NPs exhibited higher nanotoxicity effects compared to the bulk ZnO. It was observed that at a concentration of 500 mg kg^−1^, ZnO-NPs induced oxidative stress in leaves due to the overproduction (around 61% higher) of H_2_O_2_ as compared to the control or bulk ZnO treatment. These NPs also caused a reduction in the level of CAT and APOX, the free radical scavenging enzymes, in leaves and roots. Further, the level of Thiobarbituric Reactive Species (TBARS), a byproduct of lipid peroxidation was also found to be increased in plants treated with ZnO NPs. Ultimately, the study revealed that these NPs exhibit nanotoxicity that confronts the defensive system of plants and leads to damage of lipids, proteins, and DNA. Further, a study by Okupnic et al. showed a very high level of ROS generation via the treatment of very low concentration (10 mg/L) of TiO-NPs having a size range of less than 25 nm in the *Hydrilla verticillata* plant [125]. Anjum et al. [12] attributed the toxicity effects of nanoscale copper particles (Nano-Cu) to the uncontrolled generation and less metabolism of ROS in soil-pant system.

Nano bubbles (NBs) have been found to have both synergistic and negative effects [93]. Tests with spinach and carrot seeds indicated that NBs can promote sprouting growth but high-number density NB water can also negatively affect hypocotyl elongation and chlorophyll formation. Oxygen NBs demonstrated more toxicity in gram +ve bacteria than in gram −ve bacteria [69]. These properties of NBs can be exploited beneficially in the biomedical applications. Hydroxyl radicals (OH·) have been confirmed to be the specific ROS produced by NB waters [93]. Hydrogen NB water was noted to exhibit higher scavenging of endogenous and exogenous ROS than the water without NBs [92].

## Short term and long term effects on humans and animals

Nano particles/nano formulations have not only been found to exert an untoward impact on plants and beneficial microorganisms in the soil [54] but also some nano- toxicological studies have pointed to the movement of NPs from field to crop and from crop to food [81], raising concerns about the short term and long term effects on humans and animals. Exposure of NPs used in crops to non-target humans and animals can be in several ways. For animals, these NPs have been reported to be found in sewage, aquatic and terrestrial environments where they may come in direct contact with embryos, growing animals, and adults, and can affect various important physiological mechanisms. A study done in the USA and Europe reported the sewage treatment via TiONPs, Ag NPs, CNT, Fullerenes, and Zn NPs exerting toxic effects on aquatic animals and other living organisms [51]. ZnO-NPs have been reported for their strong hepatocyte toxicity in fish due to dissolved salt released from NPs [46]. Further, AgNPs were reported to exert hepatotoxicity through oxidative stress via ROS generation [32].

For humans, inhalation, oral intake, and skin exposure can lead to various long-term side effects where these NPs accumulate in the lungs, liver, heart, kidney, spleen, alimentary tract, and cardiac muscles. TiO NPs, commonly used as a food additive, is reported to generate oxidative stress and inflammations that lead to serious long term effect via apoptosis and chromosomal instability. The food-grade NPs have also been reported to intensify the existing colon cancer [123]. Gliga et al. [50] performed a study on silver NPs for their long-term exposure effect on lung bronchial epithelial cells. The effect was analyzed via RNA sequencing and genome-wide DNA methylation analysis. The transcriptome analysis revealed 1717 genes changed their expression among which 998 genes were upregulated and the rest 719 genes were down-regulated. The change in gene expression pattern affects many natural pathways including fibrosis and epithelial to mesenchymal transition (EMT) pathway that can become a major reason for bronchogenic carcinoma. The study also did a comparative analysis of 10 nm and 75 nm-sized Ag NPs and revealed that smaller-sized NPs are more cytotoxic than larger ones.

The toxicity assessment of nanomaterials on the reproductive system and hormone secretion in animals would need long-term studies and therefore, limited literature is available. Published literature indicated that the adverse effect of nanomaterials on the reproductive health and hormone secretion in animals is based on the chemical structure, shape, size, agglomeration state, surface area, and functionalization of NPs (Table 3). For instance, it has been observed the intratracheal administration of carbon NPs (14 nm) at a dose of 1 mg has the potential to cause low sperm count in mice [173]. Similarly, ZnO NPs induced cytotoxic action on murine testicular germ cells in a dose-dependent manner has been observed [48, 182]. Similarly testicular toxicity of TiO_2_-NPs in a dose-dependent exposure manner in rats has been observed by Hong et al. [63]. Later, Xu et al. [162] mentioned that Si NPs toxicity adversely affects the maturation process of spermatozoa in the epididymis in mice by triggering oxidative stress and damaging mitochondria, and resulted in energy metabolism dysfunction. Another nanomaterial toxicity study made by Yoisungnern et al. [163] on murine sperm cells in rats indicated that Ag NPs reduces the success rate of in vitro fertilization and slow down the blastocyst formation. Lafuente et al. [87] reported that oral sub-chronic exposure of PVP-Ag NPs in rats can induce a negative influence on sperm morphology in rats, even if the exposure happens during the prepubertal stage. Further, they explained that reduction in sperm production and induced sperm lesions in exposed mouse is due to the reductions of daily food and water intake, biochemical dysfunctions, and oxidative stress. Nazar et al. [116] showed that Au NPs and Ag NPs administration for 35 days in mice resulted in a significant reduction in sperm motility and normal morphology, as well as sperm chromatin remodeling, lower chromatin stability, and increased DNA damage. Similar effects due to direct interaction of Ag NPs have also been observed in bull sperms [165]. Silver nanoparticles during prepubertal development have been noted to decrease adult reproductive parameters as well as exert cytotoxic effects on testicular tissues [103, 139]. Besides this, Au NPs also noticed to impair sperm functions and lessen sperm fertilizing ability [151]. Smith et al. [147] observed functional defects and DNA damage in male mice due to oral administration of TiO_2_ NPs. Additionally, the effect on the fertility of male offspring after maternal exposure of TiO_2_ NPs has been noticed by Kyjovska et al. [86]. Toxicity of CeO_2_ NPs has been reported to cause congestion and degeneration of seminiferous tubules and decreased sperm motility and counts and increased total sperm abnormality in mice [3]. In male rats, oral administration of nanosized TiO_2_ and Ni NPs trigger toxic effects on the reproductive system by causing marked changes in the weight of the testis and epididymis [113]. It was also noticed that male rats exposed to silver nanoparticles (AgNPs) induced alterations in the testis seminiferous tubule morphometry [94]. It has been observed that Mn_3_O_4_ NPs (~ 20 nm) significantly decrease the sperm quality of rats, resulting in a decline in fertility after repeated intravenous injection for 120 days [168]. A recent study has reported that nano-TiO_2_ can penetrate through the BTB and enter the testicular tissue, leading to damage to the testis and epididymis, and reduces sperm concentration via disrupting meiosis and related signaling pathways [62, 64].

**Table 3:** Toxicity effects of nanomaterials on the reproduction and hormone secretion by animals

Nanomaterial(s)	Tested concentrations	Test animals	Type of study	Salient experimental finings	Ref.
Anatase TiO_2_	10–100 mg kg^−1^	Mouse	In-vivo	Increase in sperm malformation and rate of sperm cell MN Decreased in germ cell number and spherospermia, interstitial glands vacuole, malalignment, and vacuolization of spermatogenic cells in mice testes Increased ROS in testicular cells Superoxide dismutase activity decreased, and the malondialdehyde content increased in the TiO_2_ NP-treated groups	Song et al. [ 174]
Carbon black NPs	0.1 mg kg^−1^	Mouse	In-vivo	Partial vacuolation of the seminiferous tubules	Yoshida et al. [ 175]
Carbon nanotubes (CNTs)	1.0 mg mL^−1^	Mouse	In-vivo	Oxidative stress and reduction in the thickness of the seminiferous epithelium in the testis	Bai et al. [173]
CdTe QDs	0.2–2 nmol per mouse	Mouse	In-vivo	Cause testes toxicity in a dose-dependent manner	Li et al. [176]
Mn_2_O_3_ NPs	100–400 ppm	Rats	In-vivo	Reduce testosterone, spermatogonial cells, primary spermatocyte, spermatid, and Leydig cell	Negahdary et al. [117]
TiO_2_	200–500 mg kg^−1^	Mouse	In-vivo	Reduce sperm count and function; induce germ cell apoptosis	Guo et al. [178]
ZnO NPs	0.04–16 µg mL^−1^	Mouse	In-vivo	Decrease GSH and MDA levels; sertoli cell membrane disfunctioning; increase in ROS production; down-regulating the expression of BTB junction proteins and cell cycle arrest at S-phase in spermatocytes	Liu et al. [93]
ZnO NPs	70 nm	Mouse	In-vitro and in-vivo	Negative effect on the spermatogenesis and male fertility through DNA damage induced by ROS synthesis	Han et al. [180]
ZnO NPs	5–300 mg kg^−1^	Mouse	In-vivo	Induced formation of multinucleated giant cells in the germinal epithelium caused a significant decrease in seminiferous tubule diameter, seminiferous epithelium height, and maturation arrest	Talebi et al. [181]
ZnO NPs	250–700 mg kg^−1^	Mouse	In-vivo	Degeneration and reduction in cell types (e.g. seminiferous tubules, spermatogonia, primary spermatocyte, spermatid and sperm cells, Leydig, fibroblast cells and blood vesicles)	Mozaffari et al. [182]
ZnO NPs	50–450 mg kg^−1^	Mouse	In-vivo	Reduction in sperms in the epididymis and the concentration of testosterone in serum	Tang et al. [183]
ZnO NPs	100–400 mg kg^−1^	Mouse	In-vivo	Decreased in sperm cell count, sperm motility, live and normal sperms, serum testosterone level; antioxidant enzymes activity and increase in lipid peroxidation	Hussein et al. [184]
Mn_3_O_4_ NPs	20 nm	Rats	In-vivo	Decrease the sperm quality of rats, resulting to the decline in fertility after repeated intravenous injection for 120 days	Zhang et al. [168]
Nano-TiO_2_	1.25–5 mg kg^−1^	Mice	In-vitro	Reductions of FSH and LH concentrations and suppression of spermatogenesis	Zhou et al. [170]
Nano-TiO_2_	10–40 g mL^−1^	Rat	In-vivo	Inhibition of testosterone production by inducing dysfunctioning of the cAMP/CGMP/EGFR/MMP signaling pathway	Hong et al. [64]

There are a limited number of reports that demonstrate the effects of nanomaterials on hormone secretion in animals. Karpenko et al. [77] reported that CeO_2_ NPs have toxic effects on sex hormones and sexual glands. The level of testosterone increased after 70 days [77]. Another study reported that AgNPs can alter the sex hormone concentration in the plasma and testes and the expression of genes involved in steroidogenesis and steroids metabolism in male rats when exposed to Ag NPs/Ag SPs [39]. The toxicity of Ag NPs has also been reported to interrupt the functions of sex hormones by reducing the number of Leydig cells and sperm parameter indices [19]. Adult male rats exposed to Mn_2_O_3_ NPs had a significant reduction in the hormones such as luteinizing hormone, follicle-stimulating hormone, and testosterone [117]. Adebayo et al. [3] observed a significant reduction in serum testosterone hormone after intraperitoneal injection of 100 μg kg^−1^ of CeO_2_ NPs to mice thrice a week for five consecutive weeks. Similar reports of reduction in sperm motility, velocity, kinematic parameters, and concentrations of luteinizing hormone, follicle-stimulating hormone, and testosterone have been noticed in male rate when exposed to AgNPs [126]. Recently, Hong et al. [64] reported that Nano-titanium dioxide (nano-TiO_2_) inhibits testosterone synthesis in male mice or rats by dysfunctioning the cAMP/CGMP/EGFR/MMP signaling pathway.

## The margin of benefits over the convention

The benefits of using nanofertilzers seem to come from not only size changes but also due to the changed mode of application (e.g. foliar application instead of soil application). It is still not very clear (due to lack of one-to-one comparisons) whether the liquid foliar fertilizers, which have nutrients as ionic species, would have the same advantage as that of nanofertilizers with nutrients as particles. Further, if the foliar feeding (of dissolved ions or NPs) has an advantage then is it significant enough to compensate for increased energy use in the production and field application of nanofertilizers keeping in mind that the total nutrient requirement of a crop may not be fulfilled by one or two rounds of nanofertilizer application to crop as used to be the case for the conventional fertilizers which have higher loads of nutrients, and often excess doses are applied? Nanoparticles have been shown to have special properties due to enhanced surface area (proportional to reduced size), and that may have significant advantages in case of chemicals/materials needed for crop production in small quantities (pesticides, micronutrients) but maybe not with fertilizer nutrients which are needed by crop in higher quantities (e.g. macronutrients). Macronutrients (N, P, K) drive most of the crop productivity but at the same time, they are also the main contributors to water and air pollution [89]. The real advantage would come from the increased efficiency of macronutrients, and with the use of nano-fertilizers for macronutrients, as reduced amounts of these nutrients only would lead to significant environmental benefits such as reduced nitrate and phosphorus pollution, and reduced N_2_O emissions. Optimization of doses in their case may be highly important because just size-based or uptake-efficiency-based assumptions may not suffice meeting crop requirements as there is a threshold amount of nutrient (especially macro) that a plant would need to meet the productivity goal. Otherwise, application of less than needed amounts might lead to the mining of that macro-nutrient from the soil unless supply comes from a complimentary source such as biological fixation or in-situ crop residue recycling or ex-situ application of organic matter/ manures. The cost of new technology, for production at the industrial scale, can also be a roadblock and may delay switching to nanofertilizers [67].

Most nanotoxicity and genotoxicity related studies have involved metal oxide NPs, and only a few studies involved NPs of elements that fall in the crop micronutrient category (e.g. Zn, Mn, and Cu). Yet, these are not the typical salts that are used as fertilizers for crop production. For nanofertilizer industry and research, the focus should be on salts which are commonly used as fertilizers. Then only one to one comparison of nanoforms and conventional forms can be done. For example, most micronutrient fertilizers are sulfate salts (ZnSO_4_, FeSO_4_, CuSO_4_, etc.). Metal oxides are rarely used as fertilizers. There are also pieces of evidence that establish that polymer stabilization significantly reduces toxicity compared to the non-coated NPs [27]. Few other studies also revealed that the polymeric nanoformulations themselves may not exert any kind of toxicity and therefore it is acceptable and better to move with the biodegradable and safe polymeric NPs. At the same time, a few other studies point to the negative impacts of polymeric NPs as well. A study by Khalifa et al*.* [78] revealed the negative impact of CS-PMAA-NPK NPs on root elongation, and starch accumulation at the root tip. Under this study, even the lowest concentration of the formulated NPs exerted complete mitotic arrest in the root tip cells of *Pisum sativum*. The results of the comet test assay revealed the genotoxic effect of the nanoformulation at all tested concentrations. Therefore, sufficient toxicological studies need to be undertaken to substantiate their safety for field use. Yet unlike the biomedical field where one or two species (test animals) are considered for the trial, in agricultural systems, there are more than 7000 cultivated plant species with different physiologies. Testing responses of all these plant species to a nanofertilizer would be a daunting task to deal with perfection, and therefore protocols common to all crops and species need to be developed. Added to that, the parameters like the base materials, fabrication method, size range, morphology of particles, functional groups, coatings agents, and the way of application of the nanomaterials also affect the interactions and the outcome and therefore add more complexity [131]. Yet another major concern regarding the use of nano-fertilizers is the buildup of NPs in plant tissues without transformation and assimilation or even physical entrapment on plant surfaces. This is especially important for crops where fleshy, succulent, and leafy parts are consumed such as vegetables. It may not be as big a concern in grain crops wherein most of the fertilizer application takes place much before the grain formation, and the vegetative parts (e.g. straw) are not commonly consumed by human beings. Larue et al. [88] noticed entrapment of Ag NPs in lettuce (*Lactuca sativa*) leaf surfaces and cuticle area. Priester et al. [134] noted uptake and distribution of ZnO NPs in soybean (*Glycine Max*) plant tissues. In the same study, they even noted hampering of nitrogen fixation, an important ecosystem service in legumes, in the presence of CeO_2_ NPs. These studies indicate that the type of crop can also be a limitation for the adoption of nanofertilizers especially in the case of vegetable crops where consumable parts are directly exposed to fertilizers.

## Conclusions

The growing resource limitations in crop production, and severe environmental impacts associated with the energy-intensive modern crop production systems, particularly high-volume (often indiscriminate) fertilizer use, urgently call upon us to switch to more efficient forms of fertilizers and application methods. The current levels of nutrient use efficiency achieved for most crop production systems, worldwide, are unsustainable and unacceptable. Most of the literature regarding the nanofertilizers point to a significant reduction in the nutrient loading in fertilizer to achieve the same or higher nutrient use efficiency when the nutrients are in nano-forms. Nano fertilizers can achieve better efficiency due to a several-fold increase in surface-to-volume ratio of nano-forms of nutrients, and due to their suitability for foliar application where environmental losses are further minimized. The nutrients in the conventional forms of fertilizers are available to plants in ionic forms only after solubilization in water (soil solution). Most nutrient ions have their hydrated ionic radius between 0.07 nm (Ni^2+^) and 0.380 nm (SO_4_^2−^). The ions being 100 to 1000 times smaller than nanoparticles have higher chances of getting lost (leaching, runoff) with water or getting fixed in the clay lattice spaces, making them unavailable to plants. That is why most conventional agronomic protocols have recommendations for an excess amount of fertilizer application, than that required by plant, to compensate for losses. In this regard, nano-forms of nutrients provide better chances of retention in soil (fewer fixations due to size mismatch with lattice spaces, better entrapment in soil capillaries), and therefore more uptake by crop plants. There are also genuine concerns regarding nanotoxicity and genotoxity issues with nano formulations but most toxicity studies have either been with elements and forms related to pesticides or with salts that are not conventionally used as fertilizers in crop production. For nanofertilizer industry a better way forward should focus on macro elements (N, P, K) as a substantial environmental benefit by switching to nanofertilizers can be achieved by replacing the bulk of these nutrients. With the advancement in knowledge and technology to overcome the shortcomings in nanofertilizer production (energy use, technology expense) and adoption (unknown interactions in the environment, toxicity), the use of nanofertilizers will pave a way to arrest environmental degradation, especially in the regions with high food demand and hence intensive farming with indiscriminate fertilizer use. Reclaiming the health of the ecosystems damaged due to the bulk use of fertilizers, reviving the damaged ecosystem services, and mitigating climate change by committing to promising technologies is an urgent need of the planet. Nano-fertilizers offer the best chance, at present, to achieve this goal in the agricultural sector.

## Data Availability

The data supporting the findings in the manuscript is either available within the article or in supporting information and is freely available from the corresponding author on reasonable request.

## References

[1] Abbas Q, Yousaf B, Ullah H, Ali MU, Ok YS, Rinklebe J (2020). Environmental transformation and nano-toxicity of engineered nano-particles (ENPs) in aquatic and terrestrial organisms. Crit Rev Environ Sci Technol.

[2] Abbaspour N, Hurrell R, Kelishadi R (2014). Review on iron and its importance for human health. J Res Med Sci.

[3] Adebayo O, Akinloye O, Adaramoye O (2018). Cerium oxide nanoparticle elicits oxidative stress, endocrine imbalance and lowers sperm characteristics in testes of Balb/c mice. Andrologia.

[4] Aghdam MTB, Mohammadi H, Ghorbanpour M (2016). Effects of nanoparticulate anatase titanium dioxide on physiological and biochemical performance of *Linum usitatissimum* (linaceae) under well-watered and drought stress conditions. Rev Bras Bot.

[5] Akbarzadeh A, Abasi E, Ghanei M, Hasanzadeh A, Panahi Y (2016). The effects of various chemicals on lung, skin and eye: a review. Toxin Rev.

[6] Akhavan O, Ghaderi E, Akhavan A (2012). Size-dependent genotoxicity of graphene nanoplatelets in human stem cells. Biomaterials.

[7] Akhavan O, Ghaderi E (2010). Toxicity of graphene and graphene oxide nanowalls against bacteria. ACS Nano.

[8] Alejandro S, Höller S, Meier B, Peiter E (2020). Manganese in plants: from acquisition to subcellular allocation. Front Plant Sci.

[9] Alemu EA, Leal Filho W, Azul AM, Brandli L (2019). Malnutrition and its implications on food security. Zero hunger.

[10] Alsharef J, Taha MR, Firoozi AA, Govindasamy P (2016). Potential of using nanocarbons to stabilize weak soils. Appl Environ Soil Sci.

[11] Altarelli M, Ben Hamouda N, Schneider A, Berger MM (2019). Copper deficiency: causes, manifestations, and treatment. Nutr Clin Pract.

[12] Anjum NA, Adam V, Kizek R, Duarte AC, Pereira E, Iqbal M, Lukatkin AS, Ahmad I (2015). Nanoscale copper in the soil-plant system—toxicity and underlying potential mechanisms. Environ Res.

[13] Anke M, Groppel B, Kronemann H, Grün M (1984). Nickel—an essential element. IARC Sci Publ.

[14] Antonkiewicz J, Jasiewicz C, Koncewicz-Baran M, Sendor R (2016). Nickel bioaccumulation by the chosen plant species. Acta Physiol Plant.

[15] Ashkavand P, Tabari M, Zarafshar M, Tomášková I, Struve D (2015). Effect of SiO_2_ nanoparticles on drought resistance in hawthorn seedlings. For Res Pap.

[16] Aspuru K, Villa C, Bermejo F, Herrero P, López SG (2011). Optimal management of iron deficiency anemia due to poor dietary intake. Int J Gen Med.

[17] Avila DS, Puntel RL, Aschner M (2013). Manganese in health and disease. Interrelations between essential metal ions and human diseases.

[18] Aziz HMA, Hasaneen MN, Omer AM (2016). Nano chitosan-NPK fertilizer enhances the growth and productivity of wheat plants grown in sandy soil. Span J Agric Res.

[19] Baki ME, Miresmaili SM, Pourentezari M, Amraii E, Yousefi V, Spenani HR, Talebi AR, Anvari M, Fazilati M, Fallah AA (2014). Effects of silver nano-particles on sperm parameters, number of Leydig cells and sex hormones in rats. Iran J Reprod Med.

[20] Barillet S, Jugan ML, Laye M, Leconte Y, Herlin-Boime N, Reynaud C, Carrière M (2010). *In vitro* evaluation of SiC nanoparticles impact on A549 pulmonary cells: cyto-, genotoxicity and oxidative stress. Toxicol Lett.

[21] Bhardwaj AK, Hamed LM, Sharma N, Rajwar D, Meti S, Nagaraja MS (2019). Engineered polymeric and nano-materials for taming salty soils and waters used for crop production. Research developments in saline agriculture.

[22] Bhardwaj AK, Rajwar D, Basak N, Bhardwaj N, Chaudhari SK, Bhaskar S, Sharma PC (2020). Nitrogen mineralization and availability at critical stages of rice (*Oryza sativa*) crop, and its relation to soil biological activity and crop productivity under major nutrient management systems. J Soil Sci Plant Nutr.

[23] Caulfield LE, Black RE (2004). Comparative quantification of health risks: global and regional burden of disease attributable to selected major risk factors.

[24] Chai R, Ye X, Ma C, Wang Q, Tu R, Zhang L, Gao H (2019). Greenhouse gas emissions from synthetic nitrogen manufacture and fertilization for main upland crops in china. Carbon Balance Manag.

[25] Chen H (2018). Metal based nanoparticles in agricultural system: behavior, transport, and interaction with plants. Chem Speciat Bioavailab.

[26] Chen M, Zhou S, Zhu Y, Sun Y, Zeng G, Yang C (2018). Toxicity of carbon nanomaterials to plants, animals and microbes: recent progress from 2015-present. Chemosphere.

[27] Cheng Y, Yin L, Lin S, Wiesner M, Bernhardt E, Liu J (2011). Toxicity reduction of polymer-stabilized silver nanoparticles by sunlight. J Phys Chem C.

[28] Chhipa H (2017). Nanofertilizers and nanopesticides for agriculture. Environ Chem Lett.

[29] Chhowalla M (2017). Slow release nanofertilizers for bumper crops. ACS Cent Sci.

[30] Choudhary RC, Kumaraswamy R, Kumari S, Pal A, Raliya R, Biswas P, Saharan V (2017). Synthesis, characterization, and application of chitosan nanomaterials loaded with zinc and copper for plant growth and protection. Nanotechnology.

[31] Choudhary RC, Kumaraswamy R, Kumari S, Sharma S, Pal A, Raliya R, Biswas P, Saharan V (2019). Zinc encapsulated chitosan nanoparticle to promote maize crop yield. Int J Biol Macromol.

[32] Christen V, Capelle M, Fent K (2013). Silver nanoparticles induce endoplasmatic reticulum stress response in zebrafish. Toxicol Appl Pharmacol.

[33] Cota-Ruiz K, Ye Y, Valdes C, Deng C, Wang Y, Hernández-Viezcas JA, Duarte-Gardea M, Gardea-Torresdey JL (2020). Copper nanowires as nanofertilizers for alfalfa plants: understanding nano-bio systems interactions from microbial genomics, plant molecular responses and spectroscopic studies. Sci Total Environ.

[34] Das A, Das B (2019). Nanotechnology a potential tool to mitigate abiotic stress in crop plants. Abiotic and biotic stress in plants.

[35] de Baaij JHF, Hoenderop JGJ, Bindels RJM (2015). Magnesium in man: implications for health and disease. Physiol Rev.

[36] Deshpande P, Dapkekar A, Oak MD, Paknikar KM, Rajwade JM (2017). Zinc complexed chitosan/TPP nanoparticles: a promising micronutrient nanocarrier suited for foliar application. Carbohydr Polym.

[37] Dhlamini B, Paumo HK, Katata-Seru L, Kutu FR (2020). Sulphate-supplemented npk nanofertilizer and its effect on maize growth. Mater Res Express.

[38] Du Toit P, Malan A (1937). Phosphorus and calcium deficiency diseases as two ætiologically distinct entities. Nature.

[39] Dziendzikowska K, Krawczyńska A, Oczkowski M, Królikowski T, Brzóska K, Lankoff A, Dziendzikowski M, Stępkowski T, Kruszewski M, Gromadzka-Ostrowska J (2016). Progressive effects of silver nanoparticles on hormonal regulation of reproduction in male rats. Toxicol Appl Pharmacol.

[40] Elemike EE, Uzoh IM, Onwudiwe DC, Babalola OO (2019). The role of nanotechnology in the fortification of plant nutrients and improvement of crop production. Appl Sci.

[41] Emila S, Swaminathan S (2013). Role of magnesium in health and disease. J Exp Biol.

[42] Fabiano C, Tezotto T, Favarin JL, Polacco JC, Mazzafera P (2015). Essentiality of nickel in plants: a role in plant stresses. Front Plant Sci.

[43] Faisal M, Saquib Q, Alatar AA, Al-Khedhairy AA, Ahmed M, Ansari SM, Alwathnani HA, Dwivedi S, Musarrat J, Praveen S (2016). Cobalt oxide nanoparticles aggravate DNA damage and cell death in eggplant via mitochondrial swelling and NO signaling pathway. Biol Res.

[44] Faisal M, Saquib Q, Alatar AA, Al-Khedhairy AA, Hegazy AK, Musarrat J (2013). Phytotoxic hazards of NiO-nanoparticles in tomato: a study on mechanism of cell death. J Hazard Mater.

[45] FAO, IFAD, UNICEF, WFP, WHO (2020). The state of food security and nutrition in the world 2020. Transforming food systems for affordable healthy diets.

[46] Fernández-Cruz ML, Lammel T, Connolly M, Conde E, Barrado AI, Derick S, Perez Y, Fernandez M, Furger C, Navas JM (2013). Comparative cytotoxicity induced by bulk and nanoparticulated ZnO in the fish and human hepatoma cell lines PLHC-1 and Hep G2. Nanotoxicology.

[47] Fróna D, Szenderák J, Harangi-Rákos M (2019). The challenge of feeding the world. Sustainability.

[48] Gao G, Ze Y, Zhao X, Sang X, Zheng L, Ze X, Gui S, Sheng L, Sun Q, Hong J (2013). Titanium dioxide nanoparticle-induced testicular damage, spermatogenesis suppression, and gene expression alterations in male mice. J Hazard Mater.

[49] Ghosh M, Jana A, Sinha S, Jothiramajayam M, Nag A, Chakraborty A, Mukherjee A, Mukherjee A (2016). Effects of ZnO nanoparticles in plants: cytotoxicity, genotoxicity, deregulation of antioxidant defenses, and cell-cycle arrest. Mutat Res Genet Toxicol Environ Mutagen.

[50] Gliga AR, Di Bucchianico S, Lindvall J, Fadeel B, Karlsson HL (2018). RNA-sequencing reveals long-term effects of silver nanoparticles on human lung cells. Sci Rep.

[51] Gottschalk F, Sonderer T, Scholz RW, Nowack B (2009). Modeled environmental concentrations of engineered nanomaterials (TiO_2_, ZnO, Ag, CNT, fullerenes) for different regions. Environ Sci Technol.

[52] Gransee A, Führs H (2013). Magnesium mobility in soils as a challenge for soil and plant analysis, magnesium fertilization and root uptake under adverse growth conditions. Plant Soil.

[53] Group E (2004). Down on the farm: the impact of nano-scale technologies on food and agriculture.

[54] Gupta R, Xie H (2018). Nanoparticles in daily life: applications, toxicity and regulations. J Environ Pathol Toxicol Oncol.

[55] Gupta UC, Srivastava PC, Gupta SC (2011). Role of micronutrients: boron and molybdenum in crops and in human health and nutrition. Curr Nutr Food Sci.

[56] Ha NMC, Nguyen TH, Wang SL, Nguyen AD (2019). Preparation of NPK nanofertilizer based on chitosan nanoparticles and its effect on biophysical characteristics and growth of coffee in green house. Res Chem Intermed.

[57] Haleem AM (2020). Fabrication and characterization for phosphate nanofertilizer through polymer coating technique. J Eng Technol.

[58] Hashemi E, Akhavan O, Shamsara M, Rahighi R, Esfandiar A, Tayefeh AR (2014). Cyto and genotoxicities of graphene oxide and reduced graphene oxide sheets on spermatozoa. RSC Adv.

[59] Hawkesford M, Horst W, Kichey T, Lambers H, Schjoerring J, Møller IS, White P (2012). Functions of macronutrients. Marschner’s mineral nutrition of higher plants.

[60] Hazra G (2016). Different types of eco-friendly fertilizers: an overview. Sustain Environ.

[61] He A, Jiang J, Ding J, Sheng GD (2021). Blocking effect of fullerene nanoparticles (nC60) on the plant cell structure and its phytotoxicity. Chemosphere.

[62] Hong F, Li W, Ji J, Ze X, Diao E (2020). Nanostructured titanium dioxide (TiO_2_) reduces sperm concentration involving disorder of meiosis and signal pathway. J Biomed Nanotechnol.

[63] Hong F, Si W, Zhao X, Wang L, Zhou Y, Chen M (2015). TiO_2_ nanoparticle exposure decreases spermatogenesis via biochemical dysfunctions in the testis of male mice. J Agric Food Chem.

[64] Hong F, Ze X, Li L, Ze Y (2021). Nano-TiO_2_ reduces testosterone production in primary cultured leydig cells from rat testis through the cyclic adenosine phosphate/cyclic guanosine phosphate/epidermal growth factor receptor/matrix metalloproteinase signaling pathway. J Biomed Nanotechnol.

[65] Ingenbleek Y, Kimura H (2013). Nutritional essentiality of sulfur in health and disease. Nutr Rev.

[66] Ingenbleek Y (2009). Hyperhomocysteinemia is a biomarker of sulfur-deficiency in human morbidities. Clin Chem.

[67] Iqbal MA, Hasanuzzaman M, Filho MCMT, Fujita M, Nogueiraed TAR (2019). Nano-fertilizers for sustainable crop production under changing climate: a global perspective. Sustainable crop production.

[68] Iyaka YA (2011). Nickel in soils: a review of its distribution and impacts. SRE.

[69] Jannesari M, Akhavan O, Madaah Hosseini HR, Bakhshi B (2020). Graphene/CuO_2_ nanoshuttles with controllable release of oxygen nanobubbles promoting interruption of bacterial respiration. ACS Appl Mater Interfaces.

[70] Jasrotia P, Kashyap PL, Bhardwaj AK, Kumar S, Singh GP (2018). Scope and applications of nanotechnology for wheat production: a review of recent advances. Wheat Barley Res.

[71] Jiang HS, Qiu XN, Li GB, Li W, Yin LY (2014). Silver nanoparticles induced accumulation of reactive oxygen species and alteration of antioxidant systems in the aquatic plant *Spirodela polyrhiza*. Environ Toxicol Chem.

[72] Kaiser BN, Gridley KL, Ngaire Brady J, Phillips T, Tyerman SD (2005). The role of molybdenum in agricultural plant production. Ann Bot.

[73] Kanjana D (2019). Preparation and characterization of nanoformulated zinc fertilizer by using biopolymer and their effects on cotton. Int J Chem Stud.

[74] Karami Mehrian S, De Lima R (2016). Nanoparticles cyto and genotoxicity in plants: mechanisms and abnormalities. Environ Nanotechnol Monit Manag.

[75] Kardalas E, Paschou SA, Anagnostis P, Muscogiuri G, Siasos G, Vryonidou A (2018). Hypokalemia: a clinical update. Endocr Connect.

[76] Karlsson HL, Cronholm P, Hedberg Y, Tornberg M, De Battice L, Svedhem S, Wallinder IO (2013). Cell membrane damage and protein interaction induced by copper containing nanoparticles—importance of the metal release process. Toxicology.

[77] Karpenko NA, Malukin YV, Koreneva EM, Klochkov VK, Kavok NS, Smolenko NP, Pochernyaeva SS. The effects of chronic intake of nanoparticles of cerium dioxide or gadolinium ortovanadate into aging male rats. In: Proceedings of the international conference nanomaterials: applications and properties; Sumy State University Publishing. 2013.

[78] Khalifa NS, Hasaneen MN (2018). The effect of chitosan–pmaa–npk nanofertilizer on pisum sativum plants. 3 Biotech.

[79] Kiapour H, Moaveni P, Habibi D (2015). Evaluation of the application of gibbrellic acid and titanium dioxide nanoparticles under drought stress on some traits of basil (*Ocimum basilicum* L.). IJAAR.

[80] Kizilbash N, Alruwaili J, Hai A, Khachfe HM, Ambreen J (2020). Water soluble carbon nanotubes increase agricultural yields. J Biochem Biotech.

[81] Koo Y, Wang J, Zhang Q, Zhu H, Chehab EW, Colvin VL, Alvarez PJ, Braam J (2015). Fluorescence reports intact quantum dot uptake into roots and translocation to leaves of *Arabidopsis thaliana* and subsequent ingestion by insect herbivores. Environ Sci Technol.

[82] Kottegoda N, Sandaruwan C, Priyadarshana G, Siriwardhana A, Rathnayake UA, Berugoda Arachchige DM, Kumarasinghe AR, Dahanayake D, Karunaratne V, Amaratunga GA (2017). Urea-hydroxyapatite nanohybrids for slow release of nitrogen. ACS Nano.

[83] Kubavat D, Trivedi K, Vaghela P, Prasad K, Vijay Anand GK, Trivedi H, Patidar R, Chaudhari J, Andhariya B, Ghosh A (2020). Characterization of a chitosan-based sustained release nanofertilizer formulation used as a soil conditioner while simultaneously improving biomass production of *Zea mays* L.. Land Degrad Dev.

[84] Kumar S, Trivedi A (2016). A review on role of nickel in the biological system. Int J Curr Microbiol Appl Sci.

[85] Kumari S, Choudhary RC, Kumaraswamy R, Bhagat D, Pal A, Raliya R, Biswas P, Saharan V (2019). Zinc-functionalized thymol nanoemulsion for promoting soybean yield. Plant Physiol Biochem.

[86] Kyjovska ZO, Boisen AM, Jackson P, Wallin H, Vogel U, Hougaard KS (2013). Daily sperm production: application in studies of prenatal exposure to nanoparticles in mice. Reprod Toxicol.

[87] Lafuente D, Garcia T, Blanco J, Sanchez DJ, Sirvent JJ, Domingo JL (2016). Effects of oral exposure to silver nanoparticles on the sperm of rats. Reprod Toxicol.

[88] Larue C, Castillo-Michel H, Sobanska S, Cécillon L, Bureau S, Barthès V, Ouerdane L, Carrière M, Sarret G (2014). Foliar exposure of the crop *Lactuca sativa* to silver nanoparticles: evidence for internalization and changes in Ag speciation. J Hazard Mater.

[89] Ruan L, Bhardwaj AK, Hamilton SK (2016). Robertson, GP nitrogen fertilization challenges the climate benefits of cellulosic biofuels. Environ Res Lett.

[90] Linh TM, Mai NC, Hoe PT, Lien LQ, Ban NK, Hien LTT, Chau NH, Van NT (2020). Metal-based nanoparticles enhance drought tolerance in soybean. J Nanomater.

[91] Liu R, Lal R (2014). Synthetic apatite nanoparticles as a phosphorus fertilizer for soybean (*Glycine max*). Sci Rep.

[92] Liu S, Li J, Oshita S, Kamruzzaman M, Cui M, Fan W (2021). Formation of a hydrogen radical in hydrogen nanobubble water and its effect on copper toxicity in Chlorella. ACS Sustain Chem Eng.

[93] Liu S, Oshita S, Kawabata S, Makino Y, Yoshimoto T (2016). Identification of ROS produced by nanobubbles and their positive and negative effects on vegetable seed germination. Langmuir.

[94] Lopes IMD, de Oliveira IM, Bargi-Souza P, Cavallin MD, Kolc CSM, Khalil NM, Quináia SP, Romano MA, Romano RM (2019). Effects of silver nanoparticle exposure to the testicular antioxidant system during the prepubertal rat stage. Chem Res Toxicol.

[95] Lovecká P, Macůrková A, Záruba K, Hubáček T, Siegel J, Valentová O (2021). Genomic damage induced in *Nicotiana tabacum* L. plants by colloidal solution with silver and gold nanoparticles. Plants.

[96] Ma C, White JC, Dhankher OP, Xing B (2015). Metal-based nanotoxicity and detoxification pathways in higher plants. Environ Sci Technol.

[97] Ma X, Geiser-Lee J, Deng Y, Kolmakov A (2010). Interactions between engineered nanoparticles (ENPs) and plants: phytotoxicity, uptake and accumulation. Sci Total Environ.

[98] Maathuis FJ (2009). Physiological functions of mineral macronutrients. Curr Opin Plant Biol.

[99] Mala R, Celsia Arul Selvaraj R, Barathi Sundaram V, Blessina Siva Shanmuga Rajan R, Maheswari Gurusamy U (2017). Evaluation of nano structured slow release fertilizer on the soil fertility, yield and nutritional profile of *Vigna radiata*. Recent Pat Nanotechnol.

[100] Marcus Y (1988). Ionic radii in aqueous solutions. Chem Rev.

[101] Marschner P, Rengel Z (2012). Nutrient availability in soils.

[102] Martínez-Ballesta MC, Zapata L, Chalbi N, Carvajal M (2016). Multiwalled carbon nanotubes enter broccoli cells enhancing growth and water uptake of plants exposed to salinity. J Nanobiotechnol.

[103] Mathias FT, Romano RM, Kizys MM, Kasamatsu T, Giannocco G, Chiamolera MI (2015). Daily exposure to silver nanoparticles during prepubertal development decreases adult sperm and reproductive parameters. Nanotoxicology.

[104] Mazumdar H, Ahmed GU (2011). Phytotoxicity effect of silver nanoparticles on *Oryza sativa*. Int J ChemTech Res.

[105] Meena R, Kajal K, Paulraj R (2015). Cytotoxic and genotoxic effects of titanium dioxide nanoparticles in testicular cells of male wistar rat. Appl Biochem Biotechnol.

[106] Merghany M, Shahein M, Sliem M, Abdelgawad K, Radwan AF (2019). Effect of nano-fertilizers on cucumber plant growth, fruit yield and it’s quality. Plant Arch.

[107] Mikkelsen R (2018). Nanofertilizer and nanotechnology: a quick look. Better Crops Plant Food.

[108] Miller G, Senjen R. Out of the laboratory and onto our plates: Nanotechnology in food & agriculture. A report prepared for friends of the earth Australia. Friends of the Earth Europe and Friends of the Earth United States and supported by Friends of the Earth Germany Friends of the Earth Australia Nanotechnology Project, Australia. 2008. p. 1–63.

[109] Miller JO. Soil pH and nutrient availability. FS-2016; 1054:1–5. 10.13016/M2PN59.

[110] Mohanraj J, Lakshmanan A, Subramanian K (2017). Nano-zeolite amendment to minimize greenhouse gas emission in rice soil. J Environ Nanotechnol.

[111] Mohanraj J, Subramanian KS, Lakshmanan A (2019). Role of nano-fertilizer on greenhouse gas emission in rice soil ecosystem. Madras Agric J.

[112] Morari F, Vellidis G, Gay P (2011). Fertilizers. Encyclopedia of environmental health.

[113] Morgan AM, Ibrahim MA, Noshy PA (2017). Reproductive toxicity provoked by titanium dioxide nanoparticles and the ameliorative role of Tiron in adult male rats. Biochem Biophys Res Commun.

[114] Mukherjee A, Peralta-Videa JR, Bandyopadhyay S, Rico CM, Zhao L, Gardea-Torresdey JL (2014). Physiological effects of nanoparticulate ZnO in green peas (*Pisum sativum* L.) cultivated in soil. Metallomics.

[115] Naqib S, Jahan M (2017). The function of molybdenum and boron on the plants. J Agric Res.

[116] Nazar M, Talebi AR, Hosseini Sharifabad M, Abbasi A, Khoradmehr A, Danafar AH (2016). Acute and chronic effects of gold nanoparticles on sperm parameters and chromatin structure in Mice. Int J Reprod Biomed.

[117] Negahdary M, Arefian Z, Dastjerdi HA, Ajdary M (2015). Toxic effects of Mn_2_O_3_ nanoparticles on rat testis and sex hormone. J Nat Sci Biol Med.

[118] Nhan LV, Ma C, Rui Y, Liu S, Li X, Xing B, Liu L (2015). Phytotoxic mechanism of nanoparticles: destruction of chloroplasts and vascular bundles and alteration of nutrient absorption. Sci Rep.

[119] Nido PJ, Migo V, Maguyon-Detras MC, Alfafara C (2019). Process optimization potassium nanofertilizer production via ionotropic pre-gelation using alginate-chitosan carrier. MATEC Web Conf.

[120] Nielsen FH (2008). Is boron nutritionally relevant?. Nutr Rev.

[121] Nistor N, Ciontu L, Frasinariu OE, Lupu VV, Ignat A, Streanga V (2016). Acrodermatitis enteropathica: a case report. Medicine.

[122] Nkrumah PN, Echevarria G, Erskine PD, Chaney RL, Sumail S, van der Ent A (2019). Effect of nickel concentration and soil pH on metal accumulation and growth in tropical agromining ‘metal crops’. Plant Soil.

[123] Nogueira CM, de Azevedo WM, Dagli MLZ, Toma SH, de Arruda Leite AZ, Lordello ML, Nishitokukado I, Ortiz-Agostinho CL, Duarte MIS, Ferreira MA, Sipahi AM (2012). Titanium dioxide induced inflammation in the small intestine. World J Gastroenterol.

[124] Novotny JA, Peterson CA (2018). Molybdenum. Adv Nutr.

[125] Okupnik A, Pflugmacher S (2016). Oxidative stress response of the aquatic macrophyte *Hydrilla verticillata* exposed to TiO_2_ nanoparticles. Environ Toxicol Chem.

[126] Olugbodi JO, David O, Oketa EN, Lawal B, Okoli BJ, Mtunzi F (2020). Silver nanoparticles stimulates spermatogenesis impairments and hematological alterations in testis and epididymis of male rats. Molecules.

[127] Patra A, Adhikari T, Bhardwaj A (2016). Enhancing crop productivity in salt-affected environments by stimulating soil biological processes and remediation using nanotechnology. Innovative saline agriculture.

[128] Pawar K, Kaul G (2014). Toxicity of titanium oxide nanoparticles causes functionality and DNA damage in buffalo (*Bubalus bubalis*) sperm *in vitro*. Toxicol Ind Health.

[129] Bhardwaj AK (2021). Nitrogen availability and use efficiency in wheat crop as influenced by the organic-input quality under major integrated nutrient management systems. Front Plant Sci.

[130] Pennington J, Schoen S (1996). Total diet study: estimated dietary intakes of nutritional elements, 1982–1991. Int J Vitam Nutr Res.

[131] Pérez-de-Luque A (2017). Interaction of nanomaterials with plants: what do we need for real applications in agriculture?. Front Environ Sci.

[132] Prajapati BJ, Patel S, Patel RP, Ramani V (2018). Effect of zinc nano-fertilizer on growth and yield of wheat (*Triticum aestivum* L.) under saline irrigation condition. Agropedology.

[133] Prashar P, Shah S (2016). Impact of fertilizers and pesticides on soil microflora in agriculture. Sustainable agriculture reviews.

[134] Priester JH, Ge Y, Mielke RE, Horst AM, Moritz SC, Espinosa K, Gelb J, Walker SL, Nisbet RM, An Y-J, Schimel JP, Palmer RG, Hernandez-Viezcas JA, Zhao L, Gardea-Torresdey JL, Holden PA (2012). Soybean susceptibility to manufactured nanomaterials with evidence for food quality and soil fertility interruption. PNAS.

[135] Raliya R, Saharan V, Dimkpa C, Biswas P (2017). Nanofertilizer for precision and sustainable agriculture: current state and future perspectives. J Agric Food Chem.

[136] Ramírez-Rodríguez GB, Dal Sasso G, Carmona FJ, Miguel-Rojas C, Pérez-de-Luque A, Masciocchi N, Guagliardi A, Delgado-López JM (2020). Engineering biomimetic calcium phosphate nanoparticles: a green synthesis of slow-release multinutrient (NPK) nanofertilizers. ACS Appl Bio Mater.

[137] Ramírez-Rodríguez GB, Miguel-Rojas C, Montanha GS, Carmona FJ, Sasso GD, Sillero JC, Pedersen JS, Masciocchi N, Guagliardi A, Pérez-de-Luque A (2020). Reducing nitrogen dosage in triticum durum plants with urea-doped nanofertilizers. Nanomaterials.

[138] Rastogi A, Zivcak M, Sytar O, Kalaji HM, He X, Mbarki S, Brestic M (2017). Impact of metal and metal oxide nanoparticles on plant: a critical review. Front Chem.

[139] Rezazadeh-Reyhani Z, Razi M, Malekinejad H, Sadrkhanlou R (2015). Cytotoxic effect of nanosilver particles on testicular tissue: evidence for biochemical stress and Hsp70-2 protein expression. Environ Toxicol Pharmacol.

[140] Rico CM, Majumdar S, Duarte-Gardea M, Peralta-Videa JR, Gardea-Torresdey JL (2011). Interaction of nanoparticles with edible plants and their possible implications in the food chain. J Agric Food Chem.

[141] Rout GR, Sahoo S (2015). Role of iron in plant growth and metabolism. Rev Agric Sci.

[142] Saha N, Gupta SD (2017). Low-dose toxicity of biogenic silver nanoparticles fabricated by *Swertia chirata* on root tips and flower buds of *Allium cepa*. J Hazard Mater.

[143] Schwarz G, Belaidi AA (2013). Molybdenum in human health and disease. Interrelations between essential metal ions and human diseases.

[144] Sharma G, Kumar A, Devi KA, Prajapati D, Bhagat D, Pal A, Raliya R, Biswas P, Saharan V (2020). Chitosan nanofertilizer to foster source activity in maize. Int J Biol Macromol.

[145] Shaw AK, Ghosh S, Kalaji HM, Bosa K, Brestic M, Zivcak M, Hossain Z (2014). Nano-CuO stress induced modulation of antioxidative defense and photosynthetic performance of Syrian barley (*Hordeum vulgare* L.). Environ Exp Bot.

[146] Siddiqui MH, Al-Whaibi MH (2014). Role of nano-SiO_2_ in germination of tomato (*Lycopersicum esculentum* seeds mill.). Saudi J Biol Sci.

[147] Smith MA, Michael R, Aravindan RG, Dash S, Shah SI, Galileo DS (2015). Anatase titanium dioxide nanoparticles in mice: evidence for induced structural and functional sperm defects after short, but not long-, term exposure. Asian J Androl.

[148] Sun D, Hussain HI, Yi Z, Rookes JE, Kong L, Cahill DM (2018). Delivery of abscisic acid to plants using glutathione responsive mesoporous silica nanoparticles. J Nanosci Nanotechnol.

[149] Sun L, Song F, Guo J, Zhu X, Liu S, Liu F, Li X (2020). Nano-ZnO-induced drought tolerance is associated with melatonin synthesis and metabolism in maize. Int J Mol Sci.

[150] Tarafdar C, Daizy M, Alam MM, Ali MR, Islam MJ, Islam R, Ahommed MS, Aly Saad Aly M, Khan MZH (2020). Formulation of a hybrid nanofertilizer for slow and sustainable release of micronutrients. ACS Omega.

[151] Taylor U, Barchanski A, Petersen S, Kues WA, Baulain U, Gamrad L (2014). Gold nanoparticles interfere with sperm functionality by membrane adsorption without penetration. Nanotoxicology.

[152] Torabian S, Zahedi M, Khoshgoftar AH (2017). Effects of foliar spray of nano-particles of feso4 on the growth and ion content of sunflower under saline condition. J Plant Nutr.

[153] Tripathi DK, Singh S, Singh S, Mishra S, Chauhan D, Dubey N (2015). Micronutrients and their diverse role in agricultural crops: advances and future prospective. Acta Physiol Plant.

[154] Usman M, Farooq M, Wakeel A, Nawaz A, Cheema SA, ur Rehman H, Ashraf I, Sanaullah M (2020). Nanotechnology in agriculture: current status, challenges and future opportunities. Sci Total Environ.

[155] Wang J, Koo Y, Alexander A, Yang Y, Westerhof S, Zhang Q, Schnoor JL, Colvin VL, Braam J, Alvarez PJ (2013). Phytostimulation of poplars and Arabidopsis exposed to silver nanoparticles and Ag+ at sublethal concentrations. Environ Sci Technol.

[156] Wang SL, Nguyen AD (2018). Effects of Zn/B nanofertilizer on biophysical characteristics and growth of coffee seedlings in a greenhouse. Res Chem Intermed.

[157] Ward MH, Jones RR, Brender JD, De Kok TM, Weyer PJ, Nolan BT, Villanueva CM, Van Breda SG (2018). Drinking water nitrate and human health: an updated review. Int J Environ Res Public Health.

[158] Wazir SM, Ghobrial I (2017). Copper deficiency, a new triad: anemia, leucopenia, and myeloneuropathy. J Community Hosp Intern Med.

[159] Weaver CM (2013). Potassium and health. Adv Nutr.

[160] White PJ, Broadley MR (2001). Chloride in soils and its uptake and movement within the plant: a review. Ann Bot.

[161] Xiang L, Zhao HM, Li YW, Huang XP, Wu XL, Zhai T, Yuan Y, Cai QY, Mo CH (2015). Effects of the size and morphology of zinc oxide nanoparticles on the germination of Chinese cabbage seeds. Environ Sci Pollut Res.

[162] Xu Y, Wang N, Yu Y, Li Y, Li YB, Yu YB (2014). Exposure to silica nanoparticles causes reversible damage of the spermatogenic process in mice. PLoS ONE.

[163] Yoisungnern T, Choi YJ, Han JW, Kang MH, Das J, Gurunathan S (2015). Internalization of silver nanoparticles into mouse spermatozoa results in poor fertilization and compromised embryo development. Sci Rep.

[164] Yuvaraj M, Subramanian KS (2018). Development of slow release Zn fertilizer using nano-zeolite as carrier. J Plant Nutr.

[165] Zakhidov ST, Pavliuchenkova SM, Samoilov AV, Mudzhiri NM, Marshak TL, Rudoi VM (2013). Bovine sperm chromatin is not protected from the effects ultrasmall gold nanoparticles. Izv Akad Nauk Ser Biol.

[166] Zhang G, Zhou L, Cai D, Wu Z (2018). Anion-responsive carbon nanosystem for controlling selenium fertilizer release and improving selenium utilization efficiency in vegetables. Carbon.

[167] Zhang S, Nelson A, Beales PA (2012). Freezing or wrapping: the role of particle size in the mechanism of nanoparticle–biomembrane interaction. Langmuir.

[168] Zhang X, Yue Z, Zhang H, Liu L, Zhou X (2020). Repeated administrations of Mn_3_O_4_ nanoparticles cause testis damage and fertility decrease through PPAR-signaling pathway. Nanotoxicology.

[169] Zhao FJ, Tausz M, De Kok LJ (2008). Role of sulfur for plant production in agricultural and natural ecosystems. Sulfur metabolism in phototrophic organisms.

[170] Zhou Y, Ji J, Zhuang J, Wang L, Hong F (2019). Nanoparticulate TiO_2_ induced suppression of spermatogenesis is involved in regulatory dysfunction of the cAMP-CREB/CREM signaling pathway in Mice. J Biomed Nanotechnol.

[171] Zhu D, Juan W, Liao S, Liu W (2007). Relationship between plant availability of boron and the physico-chemical properties of boron in soils. Advances in plant and animal boron nutrition.

[172] Zulfiqar F, Navarro M, Ashraf M, Akram NA, Munné-Bosch S (2019). Nanofertilizer use for sustainable agriculture: advantages and limitations. Plant Sci.

[173] Bai Y, Zhang Y, Zhang J, Mu Q, Zhang W, Butch ER, Snyder SE, Yan B (2010). Repeated administrations of carbon nanotubes in male mice cause reversible testis damage without affecting fertility. Nat. Nanotechnol..

[174] Song G, Lin L, Liu L, Wang K, Ding Y, Niu Q, Mu L, Wang H, Shen H, Guo S (2017). Toxic Effects of anatase titanium dioxide nanoparticles on spermatogenesis and testicles in male mice. Pol J Environ Stud..

[175] Yoshida S, Hiyoshi K, Ichinose T, Takano H, Oshio S, Sugawara I, Takeda K, Shibamoto T (2009). Effect of nanoparticles on the male reproductive system of mice. Int J Androl..

[176] Li X, Yang X, Yuwen L, Yang W, Weng L, Teng Z, Wang L (2016). Evaluation of toxic effects of CdTe quantum dots on the reproductive system in adult male mice. Biomaterials..

[177] Negahdary M, Arefian Z, Dastjerdi HA, Ajdary M (2015). Toxic effects of Mn2O3 nanoparticles on rat testis and sex hormone. J Nat Sci Biol Med..

[178] Guo Z, Martucci N, Moreno-Olivas F, Tako E, Mahler G (2017). Titanium dioxide nanoparticle ingestion alters nutrient absorption in an in vitro model of the small intestine. Nano Impact..

[179] Liu Q, Xu C, Ji G, Liu H, Mo Y, Tollerud DJ, Gu A, Zhang Q (2016). Sublethal effects of zinc oxide nanoparticles on male reproductive cells. Toxicol In Vitro..

[180] Han Z, Yan Q, Ge W, Liu Z-G, Gurunathan S, De Felici M, Shen W, Zhang X-F (2016). Cytotoxic effects of ZnO nanoparticles on mouse testicular cells. Int J Nanomedicine..

[181] Talebi AR, Khorsandi L, Moridian M (2013). The effect of zinc oxide nanoparticles on mouse spermatogenesis. J Assist Reprod Genet..

[182] Mozaffari Z, Parivar K, Roodbari NH, Irani S (2015). Histopathological evaluation of the toxic effects of zinc oxide (ZnO) nanoparticles on testicular tissue of NMRI adult mice. Adv Studies Biol..

[183] Tang Y, Chen B, Hong W, Chen L, Yao L, Zhao Y, Aguilar ZP, Xu H (2019). ZnO Nanoparticles induced male reproductive toxicity based on the effects on the endoplasmic reticulum stress signaling pathway. Int J Nanomedicine..

[184] Hussein MM, Ali HA, Saadeldin IM, Ahmed MM (2016). Querectin alleviates zinc oxide nanoreprotoxicity in male albino rats. J Biochem Mol Toxicol..

